# MicroRNA as a potential diagnostic and prognostic biomarker in brain gliomas: a systematic review and meta-analysis

**DOI:** 10.3389/fneur.2024.1357321

**Published:** 2024-02-29

**Authors:** Fatemeh Hasani, Mahdi Masrour, Kimia Jazi, Payam Ahmadi, Saba sadat Hosseini, Victor M. Lu, Amirmohammad Alborzi

**Affiliations:** ^1^Neuroscience Research Center, Golestan University of Medical Sciences, Gorgan, Iran; ^2^Gastroenterology and Hepatology Research Center, Golestan University of Medical Sciences, Gorgan, Iran; ^3^School of Medicine, Tehran University of Medical Sciences, Tehran, Iran; ^4^Clinical Research and Development Center, Shahid Beheshti Hospital, Qom University of Medical Sciences, Qom, Iran; ^5^Student Research Committee, Faculty of Medicine, Medical University of Qom, Qom, Iran; ^6^Faculty of Medicine, Mashhad University of Medical Sciences, Mashhad, Iran; ^7^Department of Neurosurgery, University of Miami, Miami, FL, United States

**Keywords:** glioma, MicroRNAs, brain neoplasms, prognosis, diagnosis

## Abstract

**Introduction:**

Brain neoplasms and central nervous system (CNS) disorders, particularly gliomas, have shown a notable increase in incidence over the last three decades, posing significant diagnostic and therapeutic challenges. MicroRNAs (miRNAs) have emerged as promising biomarkers due to their regulatory role in gene expression, offering potential enhancements in glioma diagnosis and prognosis.

**Methods:**

This systematic review and meta-analysis, adhering to PRISMA guidelines, included 25 studies for diagnostic accuracy and 99 for prognostic analysis, published until August 27th, 2023. Studies were identified through comprehensive searches of PubMed, Web of Science, and Scopus databases. Inclusion criteria encompassed peer-reviewed original research providing sensitivity, specificity, and area under the curve (AUC) for miRNAs in glioma diagnosis, as well as survival outcomes with hazard ratios (HRs) or mean survival.

**Results and discussion:**

Meta-analysis demonstrated miRNAs’ high diagnostic accuracy, with a pooled sensitivity of 0.821 (95% CI: 0.781–0.855) and specificity of 0.831 (95% CI: 0.792–0.865), yielding an AUC of 0.893. Subgroup analysis by specimen type revealed consistent accuracy across blood, cerebrospinal fluid (CSF), and tissue samples. Our results also showed miRNAs can be potential prognostic biomarkers. miRNAs showed significant associations with overall survival (OS) (pooled HR: 2.0221; 95% CI: 1.8497–2.2105), progression-free survival (PFS) (pooled HR: 2.4248; 95% CI: 1.8888–3.1128), and disease-free survival (DFS) (pooled HR: 1.8973; 95% CI: 1.1637–3.0933) in tissue specimens. These findings underscore miRNAs’ potential as valuable biomarkers for improving glioma diagnosis and prognosis, offering insights for enhancing clinical decision-making and patient outcomes.

## 1 Introduction

Over the last three decades, there has been a notable rise in the incidence of brain neoplasms and central nervous system (CNS) disorders. Gliomas, constituting more than 50% of all brain and CNS tumors (1), are primary brain tumors originating from neuroglial stem or progenitor cells (2, 3). They represent 24.8% of all brain and other CNS tumors and are responsible for 82.4% of malignant brain tumors (4). In 2023, the American Cancer Society predicted that there will be approximately 24,810 new cases of cancer and 18,990 deaths related to CNS tumors, which are the primary causes of cancer-related deaths among individuals under the age of 20 (5). According to the 2020 Global Cancer Observatory (GLOBOCAN) report, there are an estimated 308,102 new cases of CNS and 251,329 deaths attributed to this type of cancer (6). Additionally, a 2019 statistical report by CBTRUS revealed that the average annual age-adjusted incidence rate (AAAIR) of gliomas in the United States was approximately 7.87 per 100,000 people between 2012 and 2016 (7). Despite the continued use of surgery, radiotherapy, and chemotherapy with alkylating agents as standard treatments, the diagnosis and treatment of brain tumors, especially gliomas, present significant challenges for future neurologists, neurosurgeons, and oncologists. Consequently, the imperative is to discover novel molecular targets for enhancing both glioma diagnosis and prognosis, with the potential utilization of microRNAs (miRNAs) as biomarkers.

miRNAs are small noncoding RNA molecules that control gene expression (8) and act as oncogenes or tumor suppressors in different types of cancers (9, 10). Several studies indicate that miRNAs regulate genes by binding to their 3′-untranslated regions (3’-UTR), influencing processes such as differentiation, apoptosis, proliferation, and development (11–13). Moreover, MiRNA binding sites exhibit a broader distribution, extending beyond the 3′ UTR to encompass diverse mRNA regions such as coding sequence, the 5′ UTR, and even within promoter regions (14). Binding of miRNAs to the 5′ UTR and coding regions leads to gene expression silencing, underscoring their regulatory influence on gene function (15, 16). MiRNA interactions with promoter regions have been observed to activate transcription, highlighting their diverse role in regulating gene expression across different stages (17). A growing body of evidence shows that miRNAs are abnormally expressed in several diseases, notably tumors (18, 19). Numerous studies have reported the use of miRNAs as biomarkers for diagnosing and predicting the prognosis of human gliomas (20–22).

miRNA-21 has been extensively researched and is often highly expressed in various cancers, potentially acting as a diagnostic and prognostic biomarker. High expression of miRNA-21 was notably associated with poorer survival in glioma patients (23, 24). miRNA-21 also exhibits consistent and high diagnostic accuracy in detecting glioma (25). High miR-15b expression was also associated with a significantly poorer overall survival rate in glioma patients, emerging as an independent prognostic factor (26). In another investigation miR-15b expression closely correlated with a shortened overall survival, indicating that miR-15b may serve as an intrinsic factor exerting a crucial role in the malignant progression of gliomas (27). Moreover, upregulation of miR-193b was detected in the serum, tissues, and cells of glioma patients compared to controls, with a high diagnostic accuracy for glioma. miR-193b levels were also correlated with a poorer survival (28). Also, miR-210 has been identified as a promising non-invasive biomarker for both the diagnosis and prognosis of glioma (29). However, conflicting results have emerged owing to differences in specimen type, sample size, or study design. Furthermore, glioma cells exhibit increased levels of miR-10b, miR-130a, miR-221, miR-125, and miR-9 compared with normal brain tissue. Elevated expression of miR-10b and miR-210 in gliomas has been linked to unfavorable outcomes (30, 31).

While many studies have suggested the diagnostic and prognostic potential of miRNAs in gliomas, the findings have been inconsistent and inconclusive. Therefore, the aim of this study was to assess the accuracy of miRNAs in diagnosing and predicting the prognosis of brain gliomas through a systematic review of the literature and meta-analysis of quantitative metadata.

## 2 Methods

Our study was designed in accordance with the methodology outlined in the Preferred Reporting Items for Systematic Reviews and Meta-Analyses (PRISMA) guideline (32). We registered our systematic review and meta-analysis protocol with the registration number CRD42023459785 in PROSPERO.

### 2.1 Information sources and search strategy

We carried out a thorough search for academic papers in PubMed, Web of Science (ISI), and Scopus from their inception until August 27st, 2023. We used a search query to systematically search titles and abstracts within our selected databases. The search queries are shown in Supplementary Table S1.

### 2.2 Inclusion criteria

We included all peer-reviewed original research studies that provided sensitivity, specificity, and area under the curve (AUC) measurements of miRNAs as diagnostic markers for gliomas. To assess prognostic accuracy, we conducted peer reviews of original research studies that presented survival curves for overall survival (OS), disease-free survival (DFS), progression-free survival (PFS), and recurrence-free survival (RFS), with or without hazard ratio (HR), risk ratio (RR), or mean survival, and their corresponding 95% confidence intervals (CIs) associated with miRNAs as prognostic markers for gliomas.

Both prospective and retrospective human studies were included. Eligible studies involved cancer patients and healthy participants. For diagnostic accuracy, studies should compare miRNAs against established reference controls to assess sensitivity and specificity, regardless of assay duration. We opted not to set eligibility criteria based on healthcare settings and the total number of participants in the included studies.

Studies that had not undergone peer review, were not in the English language, utilized datasets, or fell into categories such as letters, comments, reviews, case reports, and case series were considered ineligible and were therefore excluded from our analysis.

Following the elimination of duplicate entries, two authors (FH and AA) evaluated the titles and abstracts of all identified studies according to inclusion and exclusion criteria. Upon gathering studies that met the eligibility criteria, both authors separately conducted a thorough examination of the complete texts. Any disagreements or discrepancies were resolved by consensus.

### 2.3 Data extraction

Two reviewers (FH, SS) independently collected the following data for diagnosis using a standardized extraction method: author’s name, publication year, cancer type, specimen type, sample size, name of the miRNA, control group characteristics, alterations in miRNA levels in patients compared to the control group, sensitivity, specificity, area AUC along with its 95% CI, *p*-value, Positive Predictive Value (PPV), and Negative Predictive Value (NPV). To ensure accuracy, a third researcher (AA) examined the potential disparities between the data extraction files, and any differences were addressed through mutual agreements.

For prognosis, two independent reviewers (FH and PA) obtained the following data: author’s name, publication year, cancer type, specimen type, sample size, miRNA name, control group characteristics, alterations in miRNA levels, OS, DFS, PFS, RFS, HR, RR, or mean survival, *p*-value, and their corresponding 95% CIs. A third researcher (AA) examined the potential disparities between the data extraction files.

### 2.4 Quality assessment

The Newcastle-Ottawa Scale (NOS) was used to assess the quality of the included (33). This tool was designed and recommended by the Cochrane Handbook to appraise the quality of observational studies (34). In the case of cohort studies, the primary domains to be assessed consist of selection, comparability, and outcome, with potential maximum ratings of four, two, and three stars, respectively. According to this scale, a rating of ≥7 indicates a high quality. Two authors (FH and KJ) independently appraised the quality of the studies, and in the event of any discrepancies, a third author, PA, intervened to resolve the matter. The quality assessment of the included studies can be found in Supplementary Tables S3, S4.

### 2.5 Data analysis

The bivariate random effects model, which was created by Reitsma et al., was used to combine the results of studies that discussed diagnostic specificity and sensitivity. Additionally, this model calculates the summary receiver operating characteristic (sROC) curve and AUC, both of which serve as measures of diagnostic accuracy (35). For studies that only provided AUC as the reported outcome, meta-analysis was conducted using a random-effects model with the inverse variance method. The random-effects model was employed to account for the anticipated heterogeneity across the included studies.

The prognostic significance of miRNAs was reported in OS, PFS, DFS, RFS, and EFS. Meta-analysis of prognostic values was conducted using the inverse variance method with logarithmic HRs as the primary measure. HRs were divided into two categories: HRs with values less than one and HRs with values more than one. This was done because an HR of less than one implies that the examined miRNA is cancer-protective, while an HR of more than one indicates that the miRNA is cancer-promoting. This classification was performed without considering the regulatory mechanisms linked to the miRNA under investigation.

The studies were further divided into subgroups based on the specimen sample (tissue, blood, or cerebrospinal fluid [CSF]) used in the evaluation of miRNA expression as well as the combination of the specimen sample and the quality assessment outcome. A quality score of nine or higher was deemed “good quality” in studies; seven or higher was deemed “fair quality,” and less than seven was deemed “poor quality.”

The statistical analyses and visualizations were performed in R version 4.2.2 (R Core Team [2021], Vienna, Austria) using the “meta” and “mada” packages (36, 37). The standard errors of the AUCs and HRs for the meta-analysis were calculated using 95% CI. If the AUC CI was not available, the AUC value and sample size (the Hanley and McNeil method) were used to estimate the standard error (38, 39). We employed *I*^2^ and tau^2^ statistics to assess heterogeneity. Statistical significance was determined by an *I*^2^ value exceeding 50% and a *p*-value <0.05.

## 3 Results

### 3.1 Basic characteristics

**Figure 1:**
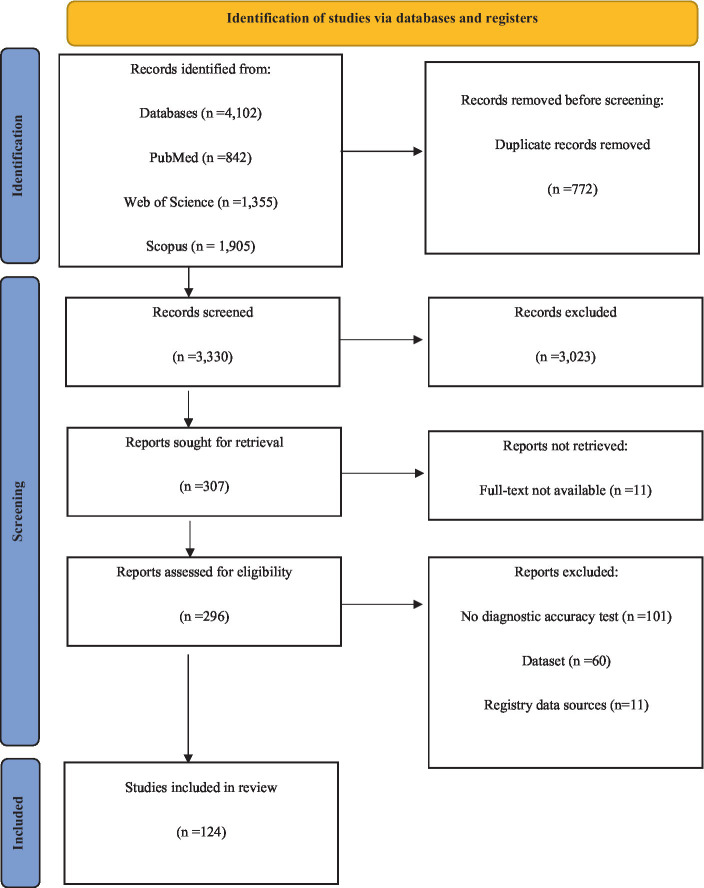
Flow chart of systematic review according to Preferred Reporting Items for Systematic Reviews and Meta-Analysis (PRISMA) guideline.

Following an initial examination of the database, a total of 4,102 titles were obtained. After the removal of duplicate articles, 3,330 articles were evaluated for inclusion. 307 full-text reviewed. 25 studies met the inclusion criteria for diagnostic accuracy, while 99 studies met the inclusion criteria for prognosis. Of the 99 articles, 58 were included in the meta-analysis. Figure 1 depicts the PRISMA flowchart, which illustrates the process of selecting and excluding the studies.

Table 1 provides a concise summary of the primary characteristics of the included studies in case of diagnosis. The basic characteristics of the included studies on prognosis are provided in Supplementary Table S2. Papers in the diagnosis segment were published between 2012 and 2022, whereas those in the prognosis section were published between 2010 and 2023. The diagnostic segment included 2,268 glioma cases and 1,334 healthy controls. To assess OS, the prognosis section analyzed 14,786 glioma cases. In addition, the PFS of 3,751 cases was evaluated. Moreover, the RFS of 443 patients from China was analyzed. We also evaluated the DFS of 460 Chinese patients.

**Table 1:** Basic characteristics of the included studies in case of diagnosis.

ID	Author, year	Country	Type of Study	Glioma type	Case samples	Control samples	Case N.	Control N.	miRNA	Down/Up Regulation	Sensitivity (%)	Specificity (%)	AUC
1	Baraniskin, 2012 (40)	Germany	Cohort	Glioma	CSF	CSF	10	10	miR-15b	upregulation	90	100	0.96
2	Wang, 2012 (41)	China	Cohort	GBM	Plasma	Plasma	50	10	miR-21	upregulation	90	100	0.93
									miR-128	upregulation	90	100	1
									miR-342-3p	upregulation	90	100	1
3	Lai, 2015 (42)	China	Cohort	Glioma	Serum	Serum	136	50	miR-210	upregulation	91.27	72.5	0.927
4	Sun, 2015 (43)	China	Cohort	Glioma	Serum	Serum	151	53	miR-128	downregulation	86.75	88.68	0.9095
5	Chai, 2015 (44)	China	Case control	Glioma	Serum	Serum	166	75	miR-199a-3p	downregulation	–	–	0.8466
6	Xiao, 2016 (45)	China	Case control	Glioma	Serum	Serum	112	54	miR-182	upregulation	58.5	85.2	0.778
7	Yue, 2016 (46)	China	Cohort	Glioma	Serum	Serum	64	45	mir-205	downregulation	86.3	92.2	0.935
8	Zhao,2016 (47)	China	Case control	Glioma	Serum	Serum	118	84	mir-451a	downregulation	81.4	79.7	0.816
9	Huang, 2017 (22)	China	Case control	Glioma	Serum	Serum	100	50	mir-376a	–	81	82	0.872
									miR-376b	–	82	78	0.89
									miR-376c	–	90	70	0.837
10	Tang, 2017 (21)	China	Case control	Glioma	Serum	Serum	74	74	miR-122	–	91.9	81.1	0.939
11	Xu, 2017 (48)	China	Case control	Glioma	Serum	Serum	47	45	miR-17	upregulation	89.3	55.3	0.78
									miR-130a	upregulation	70.2	65.2	0.72
									miR-10b	upregulation	44.6	93.6	0.72
12	Lan, 2018 (49)	China	Cohort	Glioma	Serum	Serum	60	43	miR-301a	upregulation	86.2	93.2	0.937
13	Santangelo, 2018 (50)	Italy	Case control	GBM	Serum	Serum	100	30	miR-21	upregulation	84	77	0.84
									miR-222	upregulation	57	100	0.8
									miR-124-3p	–	89	63	0.78
14	Kopkova, 2019 (51)	Czech Republic	Cohort	GBM	CSF	CSF	32	19	miR-124-3p, mir-10a, let-7b	–	73	75	0.789
15	Ohno, 2019 (52)	Japan	Cohort	Glioma	Serum	Serum	157	200	miR-4763-3p, miR-1915-3p, miR-3679-5p	upregulation	99	97	0.99
16	Wang, 2019 (53)	China	Case control	Glioma	Serum	Serum	100	100	miR-214	upregulation	90	71	0.885
17	Zhang, 2019 (54)	China	Case control	GBM	Serum	Serum	117	50	miR-145-5p	upregulation	84.6	78	0.895
18	Zhu, 2019 (28)	China	Case control	Glioma	Serum, tissues and cells	Serum, tissues and cells	112	68	miR-193b	upregulation	79.5	86.8	0.903
19	Chen, 2020 (55)	China	Case control	Glioma	Serum	Serum	122	60	miR-720	upregulation	71.3	83.3	0.773
20	Lan, 2020 (29)	China	Case control	Glioma	Serum	Serum	91	50	mir-210	upregulation	83.2	94.3	0.856
21	Sun, 2021 (56)	USA	Case control	Glioma	Serum	Serum	124	36	miR-2276-5p	downregulation	–	–	0.8107
22	Catelan, 2022 (57)	Italy	Case control	Glioma	Serum	Serum	91	30	miR-222	–	–	–	0.756
23	Geng, 2022 (58)	China	Case control	GBM	CSF	CSF	10	8	miR-9	upregulation	–	–	0.8
24	Nikolova, 2022 (59)	Bulgaria	Case control	Glioma	Brain tissue	Brain tissue	10	5	mir-21	upregulation	–	–	0.908
									mir-10b	upregulation	–	–	0.867
									mir-7	downregulation	–	–	0.861
25	Wu, 2022 (60)	Russia	Cohort	Glioma	Serum	Serum	77	85	miR-155	downregulation	66.7	76.9	0.68
									miR-410	upregulation	65.7	74.1	0.67
									miR-181a	upregulation	73.34	86	0.83
									miR-181b	upregulation	68.21	82.75	0.78
							114	85	miR-155	downregulation	82.3	84.1	0.92
									miR-410	upregulation	86.8	94.21	0.97
									miR-181a	upregulation	87.5	96.7	0.97
									miR-181b	upregulation	93.1	88.7	0.94

### 3.2 Meta-analysis of diagnostic accuracy of microRNAs in glioma

**Figure 2:**
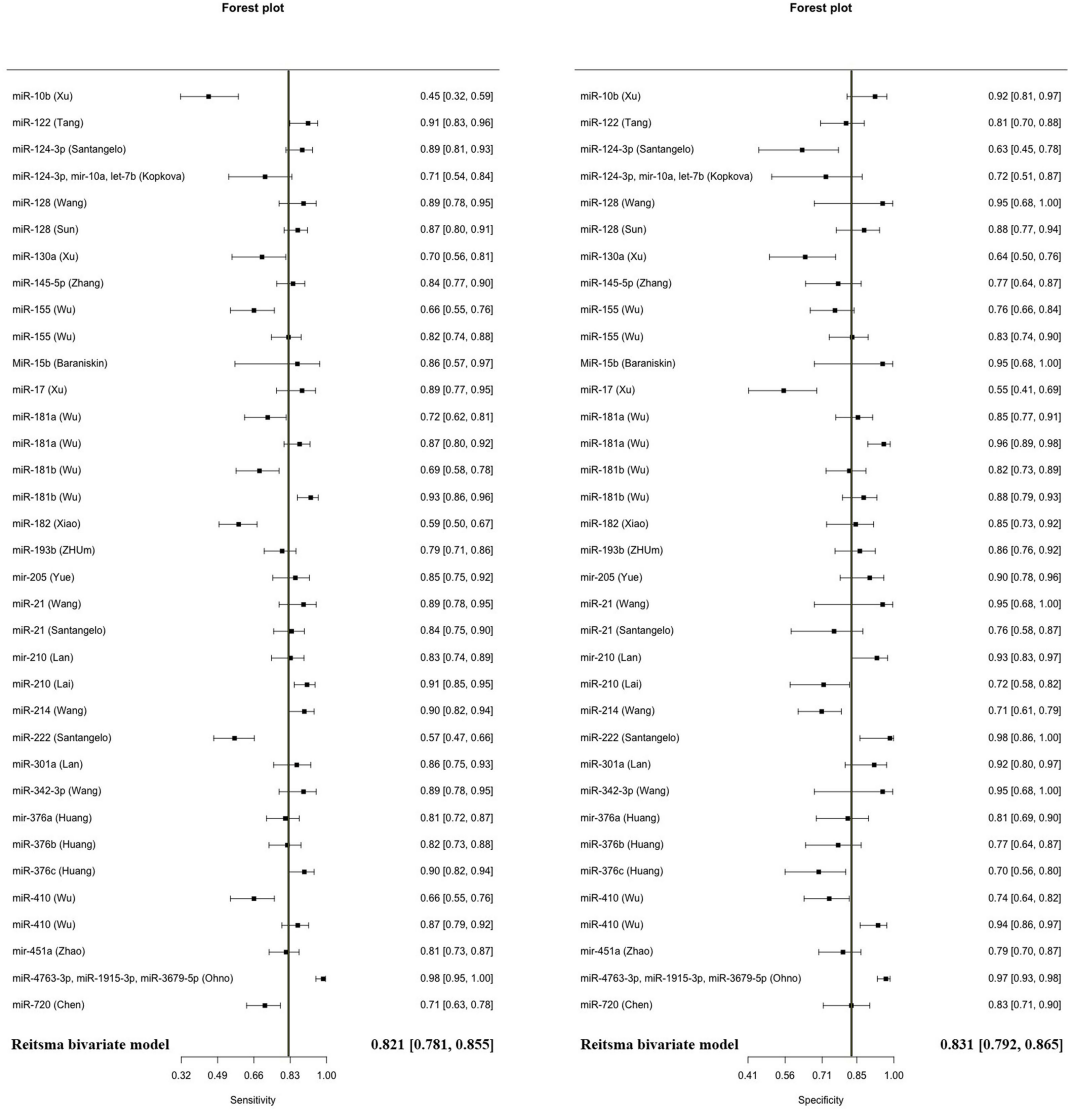
Diagnostic accuracy of microRNAs using the Reitsma bivariate model.

The model demonstrated miRNA to have a pooled sensitivity of 0.821 (95% CI: 0.781–0.855, *p* < 0.001) and a pooled specificity of 0.831 (95% CI: 0.792–0.865, *p* < 0.0001) in diagnostic evaluations involving 3,111 glioma cases and 2,045 controls (Figure 2). This analysis included 33 diagnostic evaluations using blood specimens and 2 diagnostic evaluations using CSF. The estimated *I*^2^ value using the Zhou and Dendukuri approaches was found to be 10.6%. The *p*-value for the test examining the equality of sensitivities across studies was found to be less than 2 × 10^−16^. Similarly, the *p*-value for the test assessing the equality of specificities was also less than 2e^−16^. Upon generation of the summary ROC curve (Supplementary Figure S1), the calculated AUC for all studies was determined to be 0.893. The diagnostic evaluation of glioma using microRNAs in blood specimens, consisting of 33 evaluations, yielded a cumulative sensitivity of 0.823 (95% CI: 0.782–0.857, *p* < 0.001) and a pooled specificity of 0.833 (0.792–0.866, *p* < 0.001) involving 3,069 glioma cases and 2,016 controls. The estimated *I*^2^ value using the Zhou and Dendukuri approach was found to be 12.9%. The test conducted to assess the equality of sensitivities among the blood studies yielded a *p*-value of less than 2 × 10^−16^. Similarly, the test conducted to evaluate the equality of specificities also resulted in a *p*-value of less than 2 × 10^−16^. The AUC for the blood specimen studies was 0.895. Since there have only been two studies reporting on the sensitivity and specificity of microRNAs in CSF, a meta-analysis in this specific subgroup was not conducted.

**Figure 3:**
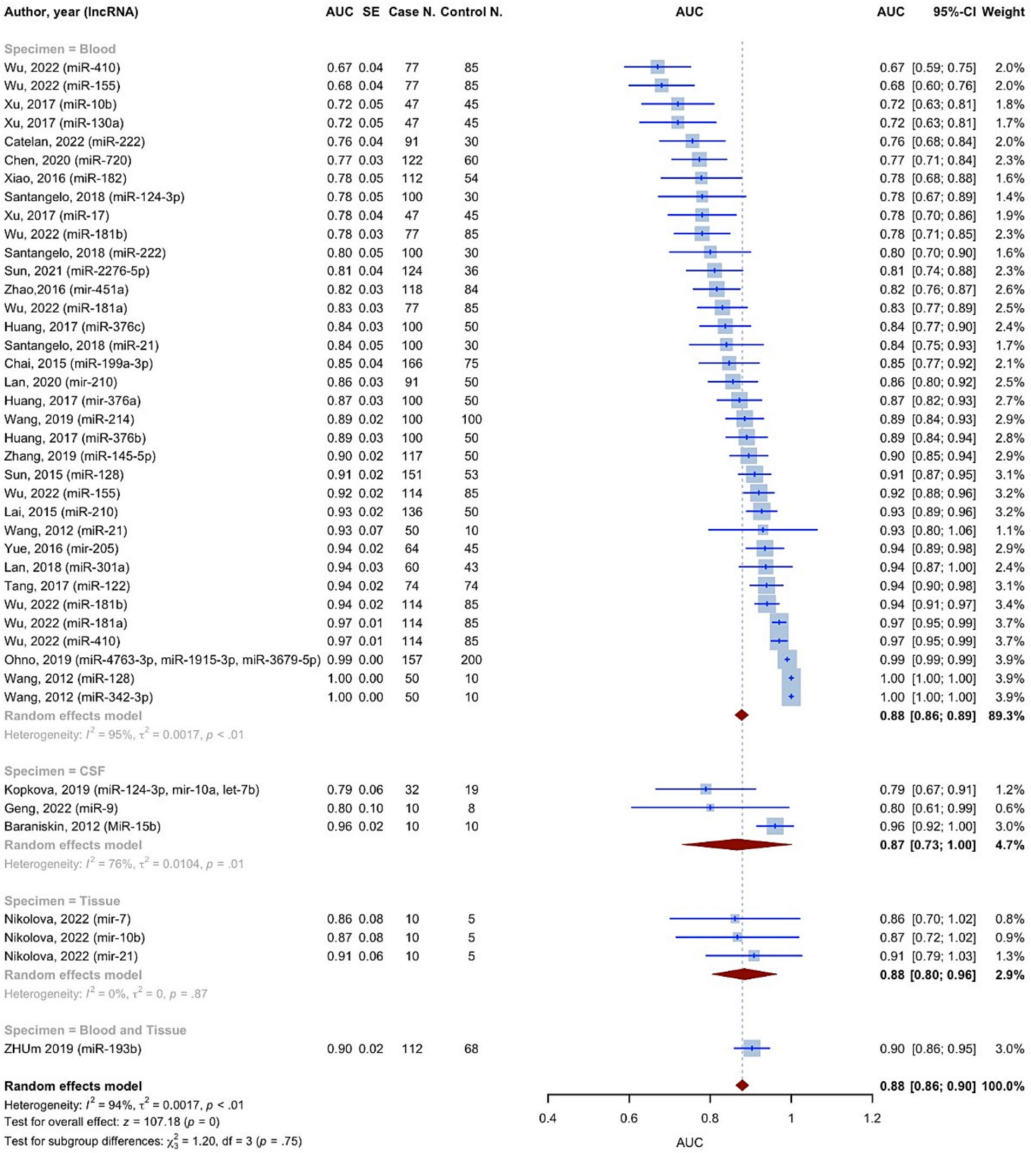
The meta-analysis of AUCs using a random effect model with inverse variance method.

The AUC values of microRNAs for detection of glioma were reported in all 42 included studies. Using the random effect model, the pooled AUC for all studies involving 3,532 glioma cases and 2,209 controls was 0.8791 (95% CI: 0.8631–0.8952, *p* < 0.0001, *I*^2^ = 94.3%) (Figure 3). The studies were divided into 4 subgroups: blood, CSF, tissue, and blood and tissue, depending on the kind of specimen used to evaluate microRNA expression. The AUC for the blood specimen subgroup with 35 evaluations involving 3,338 glioma cases and 2,089 controls was 0.8776 (95% CI: 0.8608–0.8945; *I*^2^ = 95.1%). The pooled AUC for the CSF specimen subgroup with 3 evaluations involving 52 cases and 37 controls was 0.8658 (95% CI: 0.7310–1.0007; *I*^2^ = 76.4%). The pooled AUC for the tissue specimen subgroup with 3 evaluations involving 30 cases and 15 controls was 0.8845 (95% CI: 0.8042–0.9649; *I*^2^ = 0.0%). Only one study with 112 cases and 68 controls used both blood and tissue samples simultaneously, with an AUC of 0.9030 (95% CI: 0.8596–0.9464). The test for differences between subgroups was not statistically significant (*p* = 0.7533).

Based on the findings of the quality assessment, diagnostic studies were further categorized, with quality scores of 9 and above indicating “Good quality,” 7 and above indicating “Fair quality,” and less than 7 indicating “Poor quality.” “Good quality – Blood” subgroup resulted in an AUC of 0.9051 (95% CI: 0.8825–0.9277; *I*^2^ = 95.3%) (Supplementary Figure S2).

### 3.3 Meta-analysis of the prognostic accuracy of microRNAs in glioma

**Figure 4:**
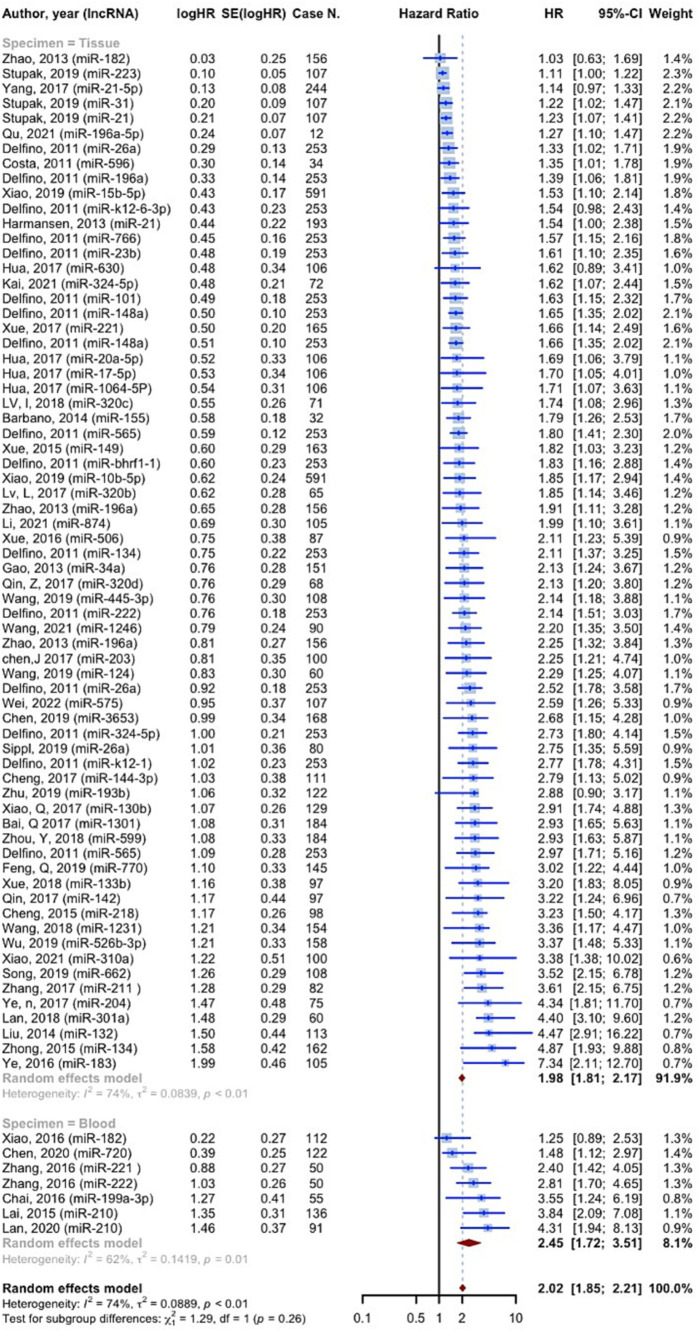
The overall survival hazard ratios meta-analysis for HRs greater than one.

**Figure 5:**
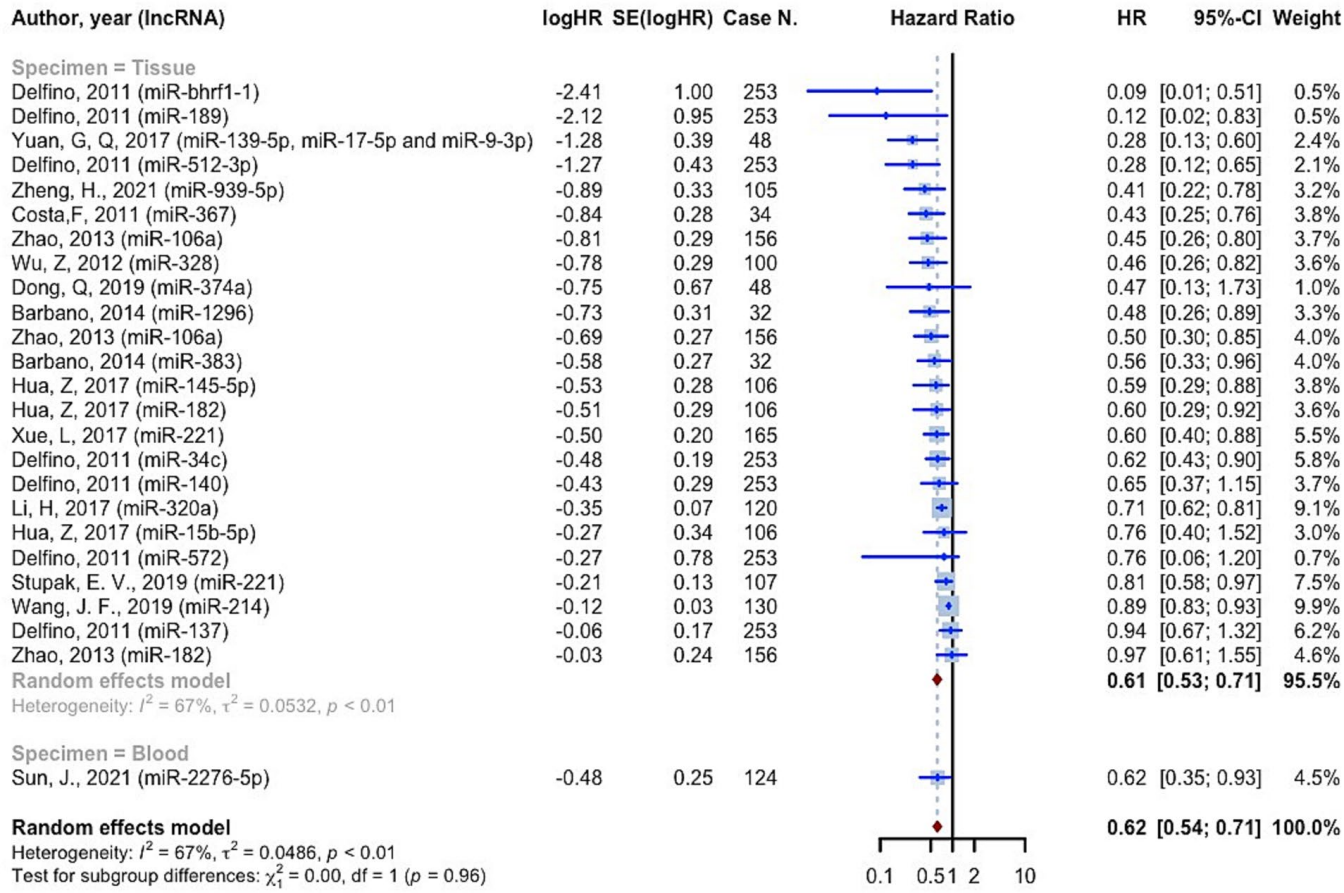
The overall survival hazard ratios meta-analysis for HRs less than one.

11,518 cases of glioma were included in 75 prognostic analyses that provided OS HRs higher than one. The overall HR for these studies was 2.0221 (95% CI: 1.8497–2.2105, *p* < 0.0001; *I*^2^ = 74.1%, *p* < 0.0001). In the tissue specimen subgroup, which included 68 evaluations, the pooled HR was 1.9836 (95% CI: 1.8106–2.1731; *I*^2^ = 73.9%). With seven evaluations, the blood specimen subgroup’s HR was 2.4547 (95% CI: 1.7178–3.5078; *I*^2^ = 62.0%). The test for differences across subgroups was not statistically significant (*p* = 0.2570) (Figure 4). 25 prognostic evaluations involving 3,602 glioma cases provided OS HRs less than one, yielding a pooled HR of 0.6154 (95% CI: 0.5366–0.7058, <0.0001; *I*^2^ = 66.7%, *p* < 0.0001). For the tissue specimen subgroup (with 24 evaluations), the pooled HR was 0.6120 (95% CI: 0.5301–0.7066; *I*^2^ = 67.5%). The blood specimen subgroup had only one evaluation with an HR of 0.6190 (95% CI: 0.3827–1.011). The test for subgroup differences was not statistically significant (*p* = 0.9645) (Figure 5).

Based on the findings of the quality assessment, diagnostic studies were further categorized, with quality scores of 9 and above indicating “Good quality,” 7 and above indicating “Fair quality,” and less than 7 indicating “Poor quality.” Quality + specimen subgrouping resulted in non-significant subgroup differences in studies with OS HRs greater than one (*p* = 0.0838). The “Good quality – Tissue” subgroup had the highest pooled HR, 3.1931 (95% CI: 1.6832–6.0574; *I*^2^ = 59.2%). The “Good quality – Blood” subgroup had HR 2.2632 (95% CI: 0.6735–7.6047; *I*^2^ = 86.6%) (Supplementary Figure S3). For studies with OS HRs less than one, quality + specimen subgrouping similarly yielded a non-significant test for subgroup differences (*p* = 0.6546). The combined HR for the “Fair quality – Tissue” subgroup was 0.6561 (95% confidence interval: 0.5407–0.7961; *I*^2^ = 72.5%) (Supplementary Figure S3).

**Figure 6:**
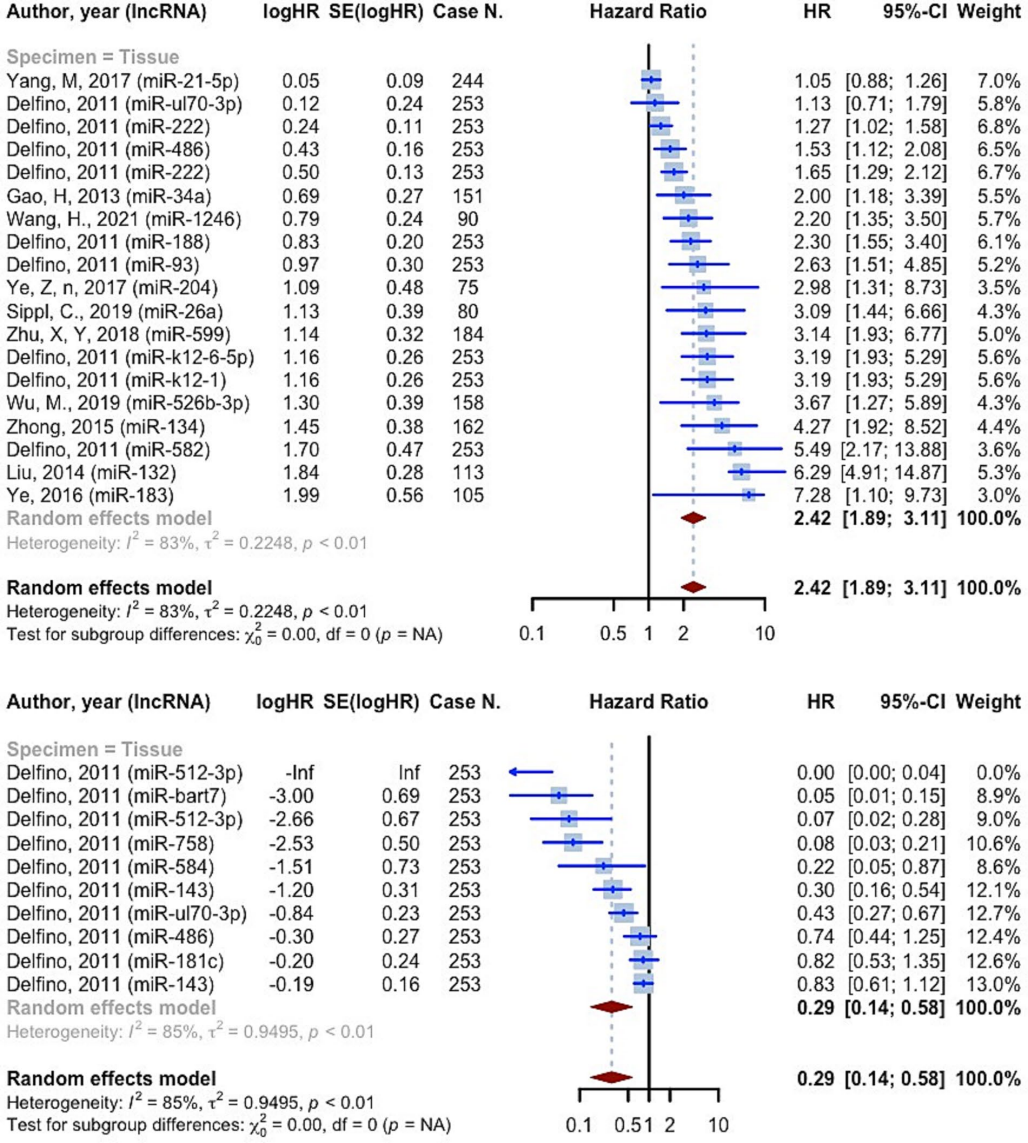
The progression-free survival hazard ratios meta-analysis.

3,639 cases of glioma were included in 19 prognostic analyses that provided PFS HRs higher than one. These evaluations were all conducted on tissue specimens. The overall HR for these studies was 2.4248 (95% CI: 1.8888–3.1128, *p* < 0.0001; *I*^2^ = 83.2%, *p* < 0.0001) (Figure 6). 10 prognostic evaluations provided PFS HR less than one, yielding a pooled HR of 0.2881 (95% CI: 0.1433–0.5791, *p* = 0.0005; *I*^2^ = 85.2%, *p* < 0.0001). These evaluations were all conducted on tissue specimens (Figure 6). Study quality subgrouping resulted in significant subgroup differences in studies with OS HRs greater than one (*p* = 0.0141). The “Fair quality” subgroup had the highest pooled HR, 4.0372 (95% CI: 2.4861–6.5561; *I*^2^ = 61.4%) (Supplementary Figure S4). All the studies with OS HRs less than one were in the “Poor quality” subgroup with pooled HR 0.2881 (95% CI: 0.1433–0.5791; *I*^2^ = 85.2%) (Supplementary Figure S4).

182 cases of glioma were included in 2 prognostic analyses that provided DFS HRs higher than one. One evaluation was on tissue specimens and one was on blood specimens. The overall HR for these studies was 1.8973 (95% CI: 1.1637–3.0933, *p* = 0.0102; *I*^2^ = 52.2%, *p* = 0.1480) (Supplementary Figure S5). 3 prognostic evaluations provided DFS HR less than one, yielding a pooled HR of 0.7816 (95% CI: 0.6479–0.9428, *p* = 0.0100; *I*^2^ = 80.9%, *p* = 0.0054). Two evaluations were on blood specimens and one on tissue specimens (Supplementary Figure S5).

### 3.4 Prognostic and diagnostic significance of individual microRNAs

**Figure 7:**
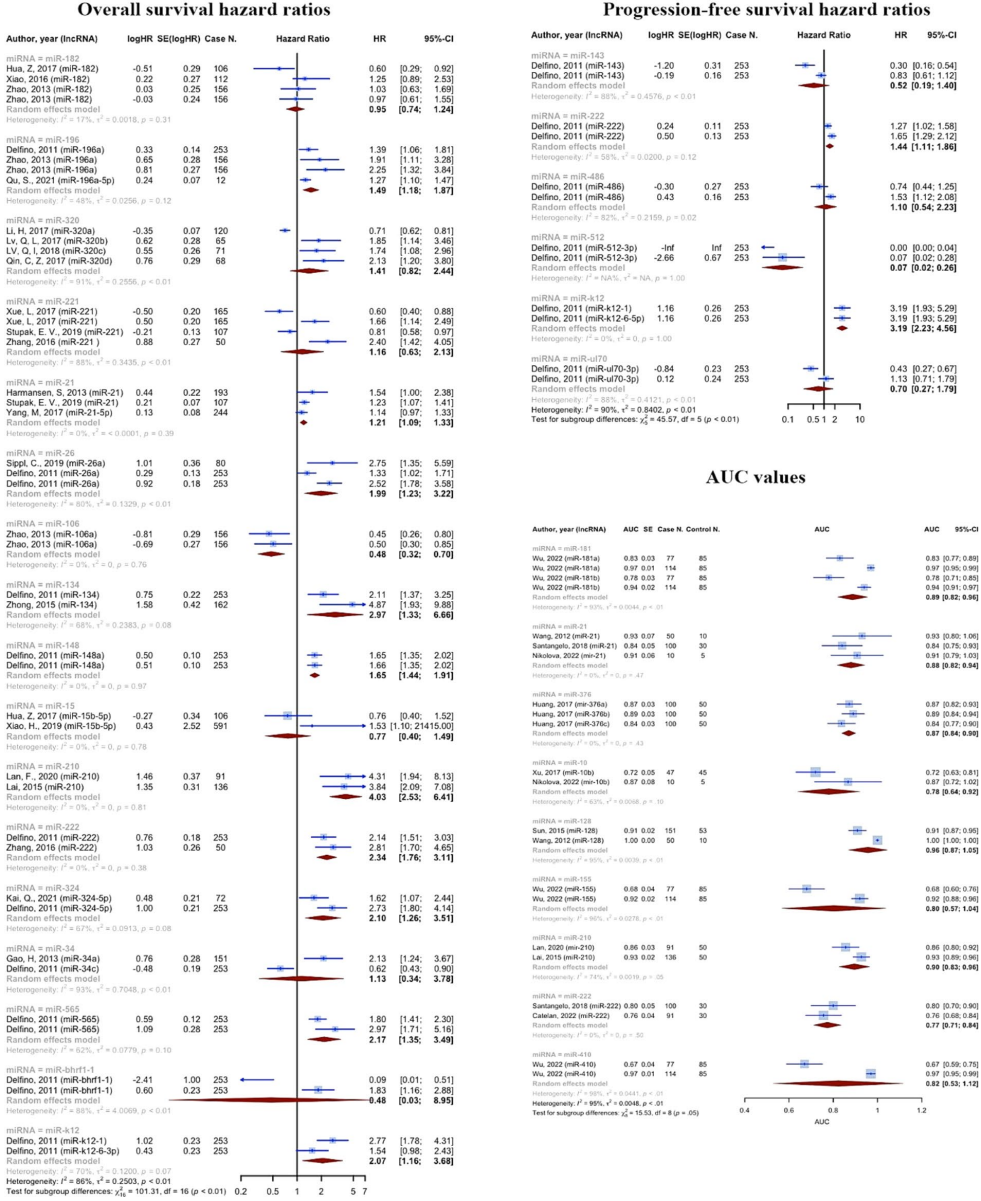
Results of the meta-analysis summarized for microRNAs with multiple evaluations.

Some microRNAs have been subjected to multiple evaluations regarding their prognostic or diagnostic accuracy. These microRNAs were analyzed individually. Table 2 and Figure 7 summarizes all of the findings.

**Table 2:** Results of the meta-analysis summarized for microRNAs with multiple evaluations.

MicroRNA	AUC evaluations	Pooled AUC [95% CI]		OS evaluations	Pooled OS HR [95% CI]		PFS evaluations	Pooled PFS HR [95% CI]	
miR-181	4	0.8875	[0.8187; 0.9564]						
miR-21	3	0.8799	[0.8166; 0.9432]	3	1.2053	[1.0902; 1.3326]			
miR-376	3	0.8701	[0.8381; 0.9020]						
miR-10	2	0.7802	[0.6385; 0.9219]						
miR-128	2	0.9570	[0.8684; 1.0456]						
miR-155	2	0.8027	[0.5676; 1.0379]						
miR-210	2	0.8957	[0.8266; 0.9648]	2	4.0310	[2.5333; 6.4140]			
miR-222	2	0.7734	[0.7107; 0.8361]	2	2.3374	[1.7554; 3.1123]	2	1.4377	[1.1128; 1.8574]
miR-410	2	0.8227	[0.5287; 1.1166]						
miR-182				4	0.9545	[0.7369; 1.2363]			
miR-196				4	1.4868	[1.1848; 1.8658]			
miR-320				4	1.4134	[0.8181; 2.4420]			
miR-221				4	1.1595	[0.6315; 2.1292]			
miR-26				3	1.9873	[1.2268; 3.2192]			
miR-106				2	0.4769	[0.3236; 0.7029]			
miR-134				2	2.9736	[1.3279; 6.6588]			
miR-148				2	1.6550	[1.4352; 1.9084]			
miR-15				2	0.7696	[0.3972; 1.4913]			
miR-324				2	2.1018	[1.2603; 3.5051]			
miR-34				2	1.1298	[0.3374; 3.7831]			
miR-565				2	2.1694	[1.3497; 3.4871]			
miR-bhrf1-1				2	0.4753	[0.0252; 8.9523]			
miR-k12				2	2.0703	[1.1646; 3.6802]	2	3.1900	[2.2334; 4.5563]
miR-143							2	0.5170	[0.1912; 1.3982]
miR-486							2	1.0983	[0.5404; 2.2319]
miR-512							2	0.0700	[0.0187; 0.2619]
miR-ul70							2	0.6964	[0.2702; 1.7950]

### 3.5 Publication bias

**Figure 8:**
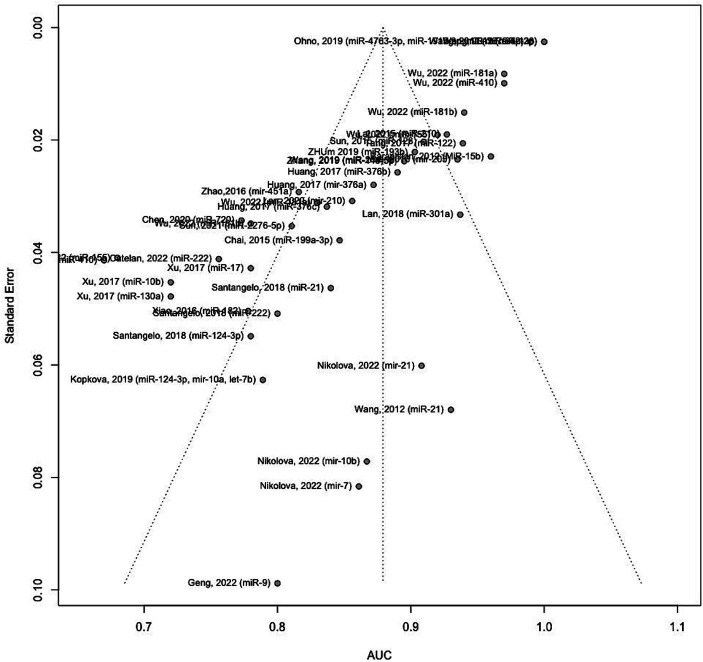
Funnel plot of included studies.

Figure 8 illustrates the funnel plot portraying the standard error for the studies included in the meta-analysis. An asymmetrical funnel plot suggests potential publication bias. To investigate this, statistical tests such as Begg’s rank correlation test and Egger’s linear regression test were employed. The results indicate significant evidence of publication bias among the included studies (Begg’s test *p*-values = 0.0908 and Eggers’ test *p*-values < 0.0001).

## 4 Discussion

### 4.1 Diagnostic value

In this study, 3,111 glioma patients and 2,045 healthy controls in 25 articles indicated a cumulative sensitivity of 0.821 (95% CI: 0.781–0.855, *p* < 0.001) and a pooled specificity of 0.831 (95% CI: 0.792–0.865, *p* < 0.0001) in diagnostic evaluations. The pooled AUC for all studies containing 3,532 glioma cases and 2,209 controls was 0.8791 (95% CI: 0.8631–0.8952, *p* < 0.0001, *I*^2^ = 94.3%). In line with our findings, Qu and colleagues indicated sensitivity, specificity, and AUC of 0.87, 0.87, and 0.93 evaluating the role of miRNAs on diagnosis of glioma, however, authors only investigated RNA profiling for the sources of heterogeneity and all different sample types from CSF, serum, and blood were investigated together (61). In this study, assessing the diagnostic accuracy, through subgroup and meta-regression analysis we divided the studies into four subgroups involving blood, CSF, tissue, and blood and tissue. The pooled AUC for each group was found 0.8776, 0.8658, 0.8845, and 0.9030, respectively. However, the difference between subgroups was not significant (*p* = 0.7533). Moreover, the diagnostic evaluation of glioma using microRNAs in blood samples, showed a cumulative sensitivity and a pooled specificity of 0.823 (95% CI: 0.782–0.857, *p* < 0.001) and 0.833 (0.792–0.866, *p* < 0.001), respectively, suggesting a remarkable diagnostic efficacy for gliomas. Similarly, He et al. explored the diagnostic value of miRNA via meta-analysis reporting overall pooled sensitivity, specificity, and AUC 0.84, 0.84, and 0.9, respectively for serum samples, considering only Asian and Chinese patients as a strong possible limitation (62). In our study, regarding limitations, this was almost resolved considering reports from Italy, Germany, China, Russia, Bulgaria, USA, Japan, and Czech Republique, thus, our findings could be more suitable for diagnostic performance compared to previous meta-analysis all around the world.

A study conducted by Lai et al. showed a reliable diagnostic efficacy of miRNA-210 with a sensitivity, specificity, and AUC of 0.9127, 0.725, 0.927 (95%CI: 0.889–0.964), respectively (42). Additionally, Sun et al. comparing glioma patients to healthy individuals, demonstrated that with an AUC of 0.9095 (95%CI: 0.8695–0.9496), miRNA-128, could promisingly distinguish glioma showing the sensitivity and specificity of 0.8675 and 0.8868, respectively (43). Qu et al. also suggested miRNA-21 with, respectively, a pooled sensitivity, specificity, and AUC of 0.82, 0.94, and 0.95 presenting a high overall diagnostic accuracy (61).

In line with previous reports, we also approved the key roles of these miRNAs in diagnosis of glioma. Our findings approved the outstanding diagnostic role of miRNA-21, miRNA-128, and miRNA-210.The first biomarker has shown antiapoptotic effects, induced tumor invasion by targeting matrix metalloproteinase, and more importantly it has been involved in chemo-and radiotherapy resistance (63). Interestingly, the majority of studies agreed on the correlation of miRNA-21, and tumor grade and aggression. Assessing fresh frozen brain tissue samples, miRNA-21, showed the lowest concentrations in grade I and II tumors, increasing by reaching higher grades peaking in GBM samples (24, 64–67). miRNA-128, known as a tumor suppressor and an favorable diagnostic biomarker in glioma (67, 68).

A recent study by Lin et al. revealed that the expression was considerably inhibited in glioma tissues, resulting in tumor cell growth and invasion by elevating COX-2 mRNA and protein expression (69). Under hypoxic situation as a major feature of the glioma microenvironment, reduction in miR-210, reduces the inhibiting effects of this miRNA on Bcl-2 19 kD interacting protein (BNIP3) gene expression which results in lower oxidative stress, less mitochondrial damage, and eventually reduced cell death (70–72). We also found the remarkable diagnostic efficacy of miRNA-181a and b in addition to miRNA-376b. Previous studies has also approved that miRNA 181 family was downregulated in glioma compared to normal brain tissue (73, 74). A study demonstrated that all the subtypes of miRNA-181, particularly type c, exhibited significant decrease with glioma progression (75). Although suppressing effects in melanoma, Huang et al. previously proved the early diagnostic role of miRNA-376 a, b, and c by their aberrant expression across tumor progression (22, 76).

There also has been further reports according to the role of miRNAs in glioma diagnosis. Serum miRNA-214 previously revealed an outstanding diagnostic value (AUC: 0.885, 95%CI: 0.833–0.926), increasing gradually with the grade of glioma increase (53). Serum exosomal miR-301a and miR-454-3p in discriminating glioma patients from healthy participants has also showed high sensitivity and specificity (49, 77). In a previous study, Zhi et al. found a panel with 9 miRNAs for diagnosis of astrocytoma with an outrageous sensitivity (0.933), specificity (0.945), and AUC (0.9722, 95%CI: 0.9501–0.9942) (78). Regarding mentioned panel, and the vast variety of miRNAs with high diagnostic values, we recommend that large sample and large-scale studies to verify panels of miRNAs, could more extensively comply early diagnosis of this lethal malignancy. In addition, future studies should consider tumor grade, and the source of sample, simultaneously, in order to create standard panels, with suitable cut-off values for each stage and reach the goal of early diagnosis.

### 4.2 Prognostic value

Consistent to the present OS, PFS, and DFS analysis, a recent meta-analysis on prognostic value of miRNAs in glioma, affirmed that both overexpression of tumor promoting miRNAs and lower expression of suppressors, are significantly associated with poor prognosis in glioma (79). 11,518 cases of glioma, included in the resent study, showed OS HRs more than one (OS HR: 2.0221 (95% CI: 1.8497–2.2105, *p* < 0.0001; *I*^2^ = 74.1%)). In the subgroup analysis considering source of sample (tissue vs. blood), we found no difference among groups. 3,639 cases of glioma provided PFS HRs of 2.4248 (95% CI: 1.8888–3.1128, *p* < 0.0001; *I*^2^ = 83.2%, *p* < 0.0001) on tissue specimens. 182 cases also showed DFS HRs of 1.8973 (95% CI: 1.1637–3.0933, *p* = 0.0102; *I*^2^ = 52.2%, *p* = 0.1480), on both tissue and blood samples. A systematic review and meta-analysis on 4,708 glioma patients, conducted by Zhang et al., revealed that the upregulation of miRNA-15b, 21, 148a, 196, 210, 221, as well as downregulation of miRNA-106a, and 124 are valuable prognostic biomarkers for poor outcomes (80). Our findings confirm the association of increased miRNA-210, and decreased miRNA-106 with poor prognosis of glioma. Noteworthy, not only advanced pathological grades, but also patients with high-grade glioma compared with low-grade, showed significantly augmented levels of miRNA-210, revealing the prognostic value of this biomarker (29, 42). However, this miRNA is known to be a biomarker for a vast variety of diseases such as cancers and cardiovascular disorders (81). This highlights the importance of assessing further miRNAs to more exclusively show the prognosis of glioma individually without being influenced by other underlying disease. Moreover, panels comprising both highly valued diagnostic and prognostic miRNAs could lead to sooner diagnosis, prognosis assessment, and therapeutic approach via a non-invasive method which could simply identify tumors not distinguished by imaging techniques.

There also have been individual studies. A meta-analysis on the cancer genome atlas glioma confirmed that overexpression of miRNA-21 is correlated with poorer OS (HR = 1.27, 95% CI: 1.01, 1.59) and PFS (HR = 1.46, 95% CI: 1.17, 1.82) (23). Although poorer OS and PFS was approved for patients with III-IV grades, the results of multivariate Cox regression analysis of covariates, conversely, demonstrated that miRNA-21 was a potential prognostic biomarker for glioma independent of known associated factors such as age, and grade (23, 82). Our data also highlighted the correlation of miRNA-222 and poor prognosis, consistently, Song and colleagues, showed the association disregarding the tumor stage (HR of IV stage patients = 1.47; 95% CI, 1.11–1.94; *p* = 0.01; HR of I–IV stage patients = 2.53; 95% CI, 1.76–3.63; *p* = 0.82) (83). Higher levels of miRNA-193b (28), miRNA-155 (84), and miRNA-222 (83) were also strongly associated with poor glioma prognosis. However, although promising diagnostic value, miRNA-130b has shown no correlation with tumor invasion and progression, and OS/DFS in gliomas (85). There is no previously reported predictive panels. In addition, there is yet controversies whether prognostic value of miRNAs is influenced by the grade of glioma, which has to be furtherly assessed. Furthermore, future research should also evaluate miRNAs with high prognostic values after each treatment stages in order to clarify the association of miRNA levels and therapeutic response.

In this study we resolved previously reported limitations such as geographical, and sample source subgroup analysis. Moreover, for the first time we compared the validity of miRNAs for both diagnostic and prognostic approaches and suggested that further studies focus on glioma specified miRNAs to avoid effects of other diseases on miRNA levels. Additionally, it is necessary to develop a novel diagnostic and prognostic panel to sooner identify small tumors that are undetectable by imaging techniques, or address patients who are at high risk of imaging and surgery. We recommend that these panels could promisingly change the future early diagnosis, prognosis, and early treatment of glioma, ameliorating patients’ morbidity and mortality rates. Although novel and positive aspects of our study, there are limitations worth to mention. First, number of available studies considering some miRNAs is limited, restricting definite conclusion. Second, some study population sizes were small. We require more high-quality studies with large populations to confirm our findings. Third, as mentioned, the prognostic and diagnostic value of miRNA biomarkers are significantly associated with tumor grade, a potential heterogenetity underlining the need of more detailed studies. Fourth, the miRNAs that has been discussed to date, are general tumor suppressors and promoters. There have been no reports of a glioma specific miRNA. Thus, further studies should focus on finding novel miRNAs or glioma specific panels exclusively, in order to differentiate glioma from other cancers, and improve the clinical application of miRNAs. Fifth, the prognostic reports were based on different indicators such as OS, DFS, and PFS, which could be the source of heterogeneity. Sixth, the included studies were all retrospective, deeming the lack of high-quality follow-up trials. Additionally, although different population were included from all around the world, still Chinese comply the majority. Last, but not least, there was a lack of cut-off value definition and difference among studies, which highlights the need for further exclusive evaluation to avoid future contradictory results.

## 5 Conclusion

In conclusion, miRNAs could be promising, non-invasive methods to both diagnose and predict the tumor invasion. Further studies should focus on the source of sample, grade of glioma, different populations as well as different miRNA detection methods, and cut off values, to provide clinically, fast response prognostic and diagnostic panels.

## Data availability statement

The original contributions presented in the study are included in the article/Supplementary material, further inquiries can be directed to the corresponding author.

## Author contributions

FH: Conceptualization, Data curation, Formal analysis, Investigation, Methodology, Software, Validation, Visualization, Writing – original draft, Writing – review & editing. MM: Conceptualization, Data curation, Formal analysis, Investigation, Methodology, Software, Validation, Visualization, Writing – original draft, Writing – review & editing. KJ: Validation, Visualization, Writing – original draft. PA: Data curation, Validation, Visualization, Writing – original draft. SH: Data curation, Validation, Visualization, Writing – original draft. VL: Supervision, Writing – review & editing. AA: Methodology, Supervision, Writing – review & editing.
