# Supplementary material for: MicroRNA as a potential diagnostic and prognostic biomarker in brain gliomas: a systematic review and meta-analysis
Source: Front Neurol. 2024 Feb 29;15:1357321. doi: 10.3389/fneur.2024.1357321 (PMC10937740; doi:10.3389/fneur.2024.1357321)
Supplement: Supplementary file 2 [file Data_Sheet_2.zip › Data Sheet 3.DOCX]

Records identified from: Databases (n =4,102)

PubMed (n =842)

Web of Science (n =1,355)

Scopus (n = 1,905)

Embase (n = x)

Records removed before screening:

Duplicate records removed

(n =772)

Records screened

(n =3,330)

Records excluded

(n =3,023)

Reports sought for retrieval

(n =307)

Reports not retrieved:

Full-text not available (n =11)

Reports assessed for eligibility

(n =296)

Reports excluded:

No diagnostic accuracy test (n =101)

Dataset (n =60)

Registery data sources (n=11)

Studies included in review

(n =124)

**Identification of studies via databases and registers**

**Identification**

**Screening**

**Included**
