# Supplementary material for: MicroRNA as a potential diagnostic and prognostic biomarker in brain gliomas: a systematic review and meta-analysis
Source: Front Neurol. 2024 Feb 29;15:1357321. doi: 10.3389/fneur.2024.1357321 (PMC10937740; doi:10.3389/fneur.2024.1357321)
Supplement: Supplementary file 2 [file Data_Sheet_2.zip › Data Sheet 2.DOCX]

Supplementary Material

| Database | Syntax | Results |
| --- | --- | --- |
| PubMed | (((((Glioma*[Title.Abstract]) OR (glial cell tumor*[Title.Abstract])) OR (mixed Glioma*[Title.Abstract])) OR (malignant Glioma[Title.Abstract])) AND (((((((diagnosis[Title.Abstract]) OR (diagnoses[Title.Abstract])) OR (postmortem diagnosis[Title.Abstract])) OR (postmortem diagnoses[Title.Abstract])) OR (antemortem diagnosis[Title.Abstract])) OR (antemortem diagnoses[Title.Abstract])) OR (prognos*[Title.Abstract]))) AND (((((((((((microRNA*[Title.Abstract]) OR (miRNA*[Title.Abstract])) OR (micro RNA[Title.Abstract])) OR (primary microRNA[Title.Abstract])) OR (primary miRNA[Title.Abstract])) OR (pri-miRNA[Title.Abstract])) OR (pri miRNA[Title.Abstract])) OR (stRNA[Title.Abstract])) OR (small temporal RNA[Title.Abstract])) OR (pre-miRNA[Title.Abstract])) OR (pre miRNA[Title.Abstract])) | 842 |
| Web of Science | TS=(“Glioma*” OR “glial cell tumor*” OR “mixed Glioma*” OR “malignant Glioma”) AND TS=(“diagnosis” OR “diagnoses” OR “postmortem diagnosis” OR “postmortem diagnoses” OR “antemortem diagnosis” OR “antemortem diagnoses” OR “prognos*”) AND TS=(“microRNA*” OR “miRNA*” OR “micro RNA” OR “primary microRNA” OR “primary miRNA” OR “pri-miRNA” OR “pri miRNA” OR “stRNA” OR “small temporal RNA” OR “pre-miRNA” OR “pre miRNA”) | 1,355 |
| Scopus | TITLE-ABS-KEY(“Glioma*” OR “glial cell tumor*” OR “mixed Glioma*” OR “malignant Glioma”) AND TITLE-ABS-KEY(“diagnosis” OR “diagnoses” OR “postmortem diagnosis” OR “postmortem diagnoses” OR “antemortem diagnosis” OR “antemortem diagnoses” OR “prognos*”) AND TITLE-ABS-KEY(“microRNA*” OR “miRNA*” OR “micro RNA” OR “primary microRNA” OR “primary miRNA” OR “pri-miRNA” OR “pri miRNA” OR “stRNA” OR “small temporal RNA” OR “pre-miRNA” OR “pre miRNA”) | 1,905 |
| Total = 4,102  After deleting duplicated files = 3,330 | |  |

# Supplementary Table 1. The complete search algorithms for PubMed, Web of Sciences, and Scopus library are as follows.

# Supplementary Table 2. Basic characteristics of the included studies in case of Prognosis.

| ID | Author, year | Country | Glioma type | Specimen | Control type | Case N. | Control N. | miRNA | Up.down regulation | | OS | PFS | RFS | DFS |
| --- | --- | --- | --- | --- | --- | --- | --- | --- | --- | --- | --- | --- | --- | --- |
| 1 | Jiang,L, 2010 | China | Astrocytomas | Brain tissue |  | 119 |  | miR-182 | up | |  |  |  |  |
|  |  |  |  |  |  |  |  | miR-183 | up | |  |  |  |  |
| 2 | Costa,F, 2011 | USA | Ependymomas | Brain tissue | Normal Brain tissue | 34 | 8 | miR-596 |  | | HR: 1.35 |  |  |  |
|  |  |  |  |  |  |  |  | miR-367 |  | | HR: 0.43 |  |  |  |
| 3 | Chang, C, 2012 | China | Glioma | Brain tissue | Non-neoplastic Brain tissues | 128 | 10 | mir-375 | down | | RR: 8.8 |  |  |  |
| 4 | Lu, S, 2012 | China | Glioma | Brain tissue | Normal Brain tissue | 108 | 20 | miR-17 | up | | RR: 5.1 |  |  |  |
| 5 | Wu, Z, 2012 | China | Glioma | Brain tissue |  | 100 |  | MiR-328 |  | | HR: low: 1, high: 0.46 |  |  |  |
| 6 | Gao, H, 2013 | China | Glioma | Brain tissue | Normal Brain tissue | 151 | 15 | miR-34a | down | | HR: high:1, low: 2.129 | HR: high:1, low: 2.002 |  |  |
| 7 | Harmansen, S, 2013 | Denmark | GBM | Brain tissue |  | 193 |  | mir-21 | up | | HR: High.low: 1.545 |  |  |  |
| 8 | Bai, Q 2017 | China | Glioma | Brain tissue | Normal Brain tissue | 184 | 184 | mir-1301 | down | | HR:high 1, low:2.931 |  |  |  |
| 9 | chen,J 2017 | China | Glioma | Brain tissue | Normal Brain tissue | 100 | 30 | miR-203 | down | | HR: 2.255 |  |  |  |
| 10 | Cheng, z,x, 2017 | China | GBM | Brain tissue | Non-tumor tissues | 111 | 111 | miR-144-3p | down | | HR: 2.791 |  |  |  |
| 11 | Hua, Z, 2017 | USA | GBM | Brain tissue |  | 106 |  | miR-630 |  | | HR:1.62 |  |  |  |
|  |  |  |  |  |  |  |  | Mir-1064-5P |  | | HR:1.71 |  |  |  |
|  |  |  |  |  |  |  |  | miR-20a-5p |  | | HR:1.69 |  |  |  |
|  |  |  |  |  |  |  |  | miR-17-5p |  | | HR:1.70 |  |  |  |
|  |  |  |  |  |  |  |  | miR-182 |  | | HR:0.60 |  |  |  |
|  |  |  |  |  |  |  |  | miR-15b-5p |  | | HR:0.76 |  |  |  |
|  |  |  |  |  |  |  |  | miR-145-5p |  | | HR:0.59 |  |  |  |
| 12 | Jing, S, Y, 2017 | China | Glioma | Brain tissue |  | 170 |  | miR-373 | down | |  |  |  |  |
| 13 | Li, H, 2017 | China | Glioma | Brain tissue | Normal Brain tissue | 120 | 20 | miR-320a | down | | HR: 0.707 |  |  | 0.635 |
| 14 | Lv, Q, L, 2017 | China | Glioma | Brain tissue | Normal Brain tissue | 65 | 16 | miR-320b | down | | HR: 1.853 |  |  |  |
| 15 | Qin, C, Z, 2017 | China | Glioma | Brain tissue | Normal Brain tissue | 68 | 16 | miR-320d | down | | HR: 2.135 |  |  |  |
| 16 | Qin, W, 2017 | China | Glioma | Brain tissue | Normal Brain tissue | 97 | 25 | miR-142 | down | | HR: 3.218 |  |  |  |
| 17 | Tang, Y, 2017 | China | Glioma | Brain tissue | Normal Brain tissue | 74 | 74 | miR-122 | down | | RR: .006 |  |  |  |
| 18 | Xiao, Z, Q, 2017 | China | Glioma | Brain tissue | Normal Brain tissue with severe TBI and craniotomy | 129 | 20 | miR-130b | up | | HR: 2.910 |  |  |  |
| 19 | Xue, L, 2017 | China | Glioma | Brain tissue | Normal Brain tissue | 165 | 8 | miR-221 | up | | HR: 1.656 |  |  |  |
|  |  |  |  |  |  |  |  | miR-221 | down | | HR: 0.604 |  |  |  |
| 20 | Yan,H, 2017 | China | Glioma | Brain tissue | Normal Brain tissue | 70 | 12 | HULC (lncRNA highly upregulated in liver cancer) | | | HR: 0.340 |  |  |  |
| 21 | Yang, M, 2017 | China & USA | Glioma | Brain tissue |  | 244 |  | miR-21-5p | up | | HR: 1.136 | HR: 1.051 |  |  |
| 22 | Ye, Z, n, 2017 | China | Glioma | Brain tissue |  | 75 |  | miR-204 | down | | HR: 4.34 | Hr: 2.98 |  |  |
| 23 | Yuan, G, Q, 2017 | China | Glioma | Brain tissue |  | 48 |  | miR-139-5p, miR-17-5p and miR-9-3p | up | | HR: 0.278 |  |  |  |
| 24 | Zhang, J, 2017 | China | Glioma | Brain tissue |  | 82 |  | miR-211 | down | | HR: 3.614 |  |  |  |
| 25 | Chen, Y, Y, 2018 | Taiwan | GBM | Brain tissue |  | 114 |  | miR-125b-5p | up | | HR: 2.332 |  |  |  |
|  |  |  |  |  |  |  |  | miR-181d-3p | up | | HR: 3.038 |  |  |  |
|  |  |  |  |  |  |  |  | miR-221-3p | up | | HR: 2.112 |  |  |  |
|  |  |  |  |  |  |  |  | miR-222-3p | up | | HR: 1.01 |  |  |  |
|  |  |  |  |  |  |  |  | miR-224-5p | up | | HR: 0.962 |  |  |  |
| 26 | Lan, F, 2018 | China | Glioma | Brain tissue | Normal Brain tissue | 60 | 43 | miR-301a | up | | HR: 4.4 |  |  |  |
| 27 | LV, Q, l, 2018 | China | Glioma | Brain tissue | Normal Brain tissue | 71 | 16 | miR-320c | down | | HR: 1.741 |  |  |  |
| 28 | Wang, H, 2018 | China | Glioma | Brain tissue |  | 154 |  | miR-1231 | down | | HR: 3.356 |  |  |  |
| 29 | Xue, K, 2018 | China | Glioma | Brain tissue | Brain tissue | 97 | 97 | miR0133b | down | | HR: 3.197 |  |  |  |
| 30 | Zhou, X, Y, 2018 | China | Glioma | Brain tissue |  | 184 |  | miR-599 | down | | HR: 2.931 | 3.137 |  |  |
| 31 | Chen, Y, 2019 | China | Glioma | Brain tissue | Normal Brain tissues | 168 | 168 | miR-3653 | down | | HR: 2.682 |  |  |  |
| 32 | Dong, Q, 2019 | China | Glioma | Brain tissue | Normal Brain tissue | 48 | 5 | miR-374a | down | | HR: 0.472 |  |  |  |
| 33 | Feng, F, Q, 2019 | China | Glioma | Brain tissue | Normal Brain tissue | 145 | 145 | miR-770 | up | | HR: 3.018 |  |  |  |
| 34 | Sippl, C., et al. ,2019 | Germany | GBM | Brain tissue | Normal Brain tissue | 80 | 80 | miR-26a |  | | HR: 2.750 | HR: 3.091 |  |  |
| 35 | Song, Q., et al. ,2019 | China | Glioma | Brain tissue | neoplastic Brain tissue | 108 | 36 | miR-662 | down | | HR: 3.525 |  |  |  |
| 36 | Stupak, E. V., et al. (2019 | Russia | Glioma | Brain tissue | Normal Brain tissue | 107 | 107 | miR-31 | up | | HR: 1.221 |  |  |  |
|  |  |  |  |  |  |  |  | miR-21 | up | | HR: 1.228 |  |  |  |
|  |  |  |  |  |  |  |  | miR-221 | up | | HR: 0.814 |  |  |  |
|  |  |  |  |  |  |  |  | miR-223 | up | | HR: 1.106 |  |  |  |
| 37 | Wang, J. F., et al. ,2019 | China | Glioma | Brain tissue |  | 130 | 100 | miR-214 | up | | HR: 0.885 |  |  |  |
| 38 | Wang, L. Q., et al. ,2019 | China | Glioma | Brain tissue | Normal Brain tissue | 60 | 40 | miR-124 | down | | HR: 2.289 |  |  | 2.421 |
| 39 | Wang, W, 2019 | China | Glioma | Brain tissue | Normal Brain tissue | 108 | 95 | miR-445-3p | up | | HR: 2.136 |  |  |  |
| 40 | Wu, M., et al. ,2019 | China | GBM | Brain tissue |  | 158 |  | miR-526b-3p | down | | HR: 3.368 | HR: 3.675 |  |  |
| 41 | Xiao, H., et al. ,2019 | China | LGG | Brain tissue | Normal Brain tissue | 591 |  | has-miR-10b-5p | up | | HR: 1.8516 |  |  |  |
|  |  |  |  |  |  |  |  | has-miR-15b-5p | up | | HR: 1.5350 |  |  |  |
| 42 | Zhang, Y., et al. ,2019 | China | GBM | Serum |  |  |  | miR-145-5p | down | | RR: 4.72 |  |  |  |
| 43 | Zhu, M., et al. ,2019 | China | Glioma | Brain tissue | Normal Brain tissue | 122 | 8 | miR-193b | up | | HR: 2.877 |  |  |  |
| 44 | Chen, J., et al. (2020) | China | Glioma | Brain tissue | Normal Brain tissue | 60 |  | circ-0079586 | up | | HR: 1.845 |  |  |  |
| 45 | Chen, P., et al. (2020) | China | Glioma | Plasma | Plasma | 122 | 60 | miR-720 | up | | HR: 1.48 |  |  | HR: 1.47 |
| 46 | Jia, Y., et al. (2020) | China | Glioma | Brain tissue |  | 186 |  | miR-195 | up | | OR: 0.359 |  |  |  |
| 47 | Lan, F., et al. (2020) | China | Glioma | Serum | Serum | 91 | 50 | miR-210 | up | | HR: 4.31 |  |  |  |
| 48 | Morokoff, A., et al. (2020) | China | Glioma | Serum | Serum | 91 | 17 | miR-320 |  | | HR: 1.51 |  |  |  |
| 49 | Kai, Q., et al. ,2021 | China | Glioma | Brain tissue | Normal Brain tissue | 72 | 28 | miR-324-5p | down | | HR: 1.62 |  |  |  |
| 50 | Li, Y., et al. ,2021 | China | Glioma | Brain tissue | Normal Brain tissue | 105 | 105 | miR-874 | down | | HR: 1.99 |  |  |  |
| 51 | Liu, P., et al. ,2021 | China | Glioma | Brain tissue |  | 92 |  | miR-21 | up | | RR: 3.401 |  |  |  |
| 52 | Qu, S., et al. ,2021 | China | Glioma | Brain tissue |  | 12 |  | has-miR-196a-5p | up | | HR: 1.27 |  |  |  |
| 53 | Sun, J., et al. ,2021 | China | Glioma | Serum | Serum | 124 | 36 | miR-2276-5p | down | | HR: 0.619 |  |  |  |
| 54 | Wang, H., et al. ,2021 | China | Glioma | Brain tissue |  | 90 |  | miR-1246 | down | | HR: 2.2 | HR: 2.2 |  |  |
| 55 | Xiao, F., et al. ,2021 | China | Glioma | Brain tissue |  | 100 |  | mir-310a | up | | HR: 3.384 |  |  |  |
| 56 | Zheng, H., et al. ,2021 | China | Glioma | Brain tissue | Normal adjacent tissues | 105 | 105 | miR-939-5p | down | | HR: 0.411 |  |  |  |
| 57 | Wei, G., et al. (2022). | China | Glioma | Brain tissue | Normal Brain tissue | 107 | 33 | miR-575 | up | | HR: 2.589 |  |  |  |
| 58 | Delfino, 2011 | USA | GBM | Brain tissue |  | 253 |  | miR-182 |  | |  |  |  |  |
|  |  |  |  |  |  |  |  | miR-189 |  | | HR: 0.12 |  |  |  |
|  |  |  |  |  |  |  |  | miR-196a |  | | HR: 1.39 |  |  |  |
|  |  |  |  |  |  |  |  | miR-221 |  | |  |  |  |  |
|  |  |  |  |  |  |  |  | miR-222 |  | | HR:2.14 |  |  |  |
|  |  |  |  |  |  |  |  | miR-23b |  | | HR:1.61 |  |  |  |
|  |  |  |  |  |  |  |  | miR-26a |  | |  |  |  |  |
|  |  |  |  |  |  |  |  | miR-324-5p |  | | HR:2.73 |  |  |  |
|  |  |  |  |  |  |  |  | miR-34c |  | | HR:0.62 |  |  |  |
|  |  |  |  |  |  |  |  | miR-bhrf1-1 |  | |  |  |  |  |
|  |  |  |  |  |  |  |  | miR-512-3p |  | | HR: 0.28 |  |  |  |
|  |  |  |  |  |  |  |  | miR-565 |  | |  |  |  |  |
|  |  |  |  |  |  |  |  | miR-572 |  | | HR: 0.76 |  |  |  |
|  |  |  |  |  |  |  |  | miR-766 |  | | HR:1.57 |  |  |  |
|  |  |  |  |  |  |  |  | miR-k12-1 |  | | HR:2.77 |  |  |  |
|  |  |  |  |  |  |  |  | miR-k12-6-3p |  | | HR:1.54 |  |  |  |
|  |  |  |  |  |  |  |  | miR-101 |  | | HR:1.63 |  |  |  |
|  |  |  |  |  |  |  |  | miR-10b |  | |  |  |  |  |
|  |  |  |  |  |  |  |  | miR-134 |  | | HR:2.11 |  |  |  |
|  |  |  |  |  |  |  |  | miR-137 |  | |  |  |  |  |
|  |  |  |  |  |  |  |  | miR-140 |  | |  |  |  |  |
|  |  |  |  |  |  |  |  | miR-148a |  | | HR: 1.65 |  |  |  |
|  |  |  |  |  |  |  |  | miR-148a |  | | HR: 1.66 |  |  |  |
|  |  |  |  |  |  |  |  | miR-181c |  | |  |  |  |  |
|  |  |  |  |  |  |  |  | miR-188 |  | |  | HR:2.3 |  |  |
|  |  |  |  |  |  |  |  | miR-296 |  | |  |  |  |  |
|  |  |  |  |  |  |  |  | miR-bart7 |  | |  | HR: 0.05 |  |  |
|  |  |  |  |  |  |  |  | miR-486 |  | |  |  |  |  |
|  |  |  |  |  |  |  |  | miR-489 |  | |  | HR:0.04 |  |  |
|  |  |  |  |  |  |  |  | miR-ul70-3p |  | |  |  |  |  |
|  |  |  |  |  |  |  |  | miR-552 |  | |  | HR:0 |  |  |
|  |  |  |  |  |  |  |  | miR-578 |  | |  | HR:0 |  |  |
|  |  |  |  |  |  |  |  | miR-582 |  | |  | HR:5.49 |  |  |
|  |  |  |  |  |  |  |  | miR-584 |  | |  | HR: 0.22 |  |  |
|  |  |  |  |  |  |  |  | miR-758 |  | |  |  |  |  |
|  |  |  |  |  |  |  |  | miR-93 |  | |  | HR: 2.63 |  |  |
|  |  |  |  |  |  |  |  | miR-k12-1 |  | |  | HR:3.19 |  |  |
|  |  |  |  |  |  |  |  | miR-k12-6-5p |  | |  | HR: 3.19 |  |  |
|  |  |  |  |  |  |  |  | miR-106b |  | |  |  |  |  |
|  |  |  |  |  |  |  |  | miR-143 |  | |  |  |  |  |
| 59 | Srinivasan, 2011 | India | GBM | Brain tissue |  | 222 |  | miR-20a | up | | HR: 0.68 |  |  |  |
|  |  |  |  |  |  |  |  | miR-106a | up | | HR: 0.66 |  |  |  |
|  |  |  |  |  |  |  |  | miR-17-5p | up | | HR: 0.68 |  |  |  |
|  |  |  |  |  |  |  |  | miR-31 | down | | HR: 1.32 |  |  |  |
|  |  |  |  |  |  |  |  | miR-222 | down | | HR: 1.26 |  |  |  |
|  |  |  |  |  |  |  |  | miR-148a | up | | HR: 1.21 |  |  |  |
|  |  |  |  |  |  |  |  | miR-221 | down | | HR: 1.27 |  |  |  |
|  |  |  |  |  |  |  |  | miR-146b | up | | HR: 1.25 |  |  |  |
|  |  |  |  |  |  |  |  | miR-200b | up | | HR: 1.21 |  |  |  |
|  |  |  |  |  |  |  |  | miR-193a | up | | HR: 1.34 |  |  |  |
| 60 | Li, 2013 | China | Glioma | Brain tissue | Non-neoplastic Brain tissues | 128 | 128 | miR-372 | down | | RR: 4.37 |  |  |  |
| 61 | Lu, 2013 | China | Glioma | Brain tissue | Normal Brain tissues | 108 | 20 | miR-224 | up | | RR:4.6 |  |  | RR:4.5 |
| 62 | Parker, 2013 | Australia | GBM | Brain tissue | Normal Brain tissues | 43 | 43 | miR-132 | up | | median: 13m |  |  |  |
| 63 (ISH) | Zhao, 2013 | China | GBM | Brain tissue |  | 156 |  | MiR-106a | down | | HR (high vs. low): 0.447 |  |  |  |
|  |  |  |  |  |  |  |  | MiR-196a |  | | HR (high vs. low): 1.906 |  |  |  |
|  |  |  |  |  |  |  |  | MiR-182 |  | | HR (high vs. low): 1.032 |  |  |  |
| 63 (PCR) | Zhao, 2013 | China | GBM | Brain tissue |  | 156 |  | MiR-106a | down | | HR (high vs. low): 0.504 |  |  |  |
|  |  |  |  |  |  |  |  | MiR-196a |  | | HR (high vs. low): 2.252 |  |  |  |
|  |  |  |  |  |  |  |  | MiR-182 |  | | HR (high vs. low): 0.974 |  |  |  |
| 64 | Barbano, 2014 | Italy | Glioma | Brain tissue | Brain tissue | 32 | 4 | miR-155 | up | | HR: 1.79 |  |  |  |
|  |  |  |  |  |  |  |  | miR-383 | up | | HR: 0.56 |  |  |  |
|  |  |  |  |  |  |  |  | miR-1296 | up | | HR: 0.48 |  |  |  |
| 65 | Lai, 2014 | China | Glioma | Brain tissue | Normal Brain tissue | 125 | 10 | miR-210 | up | | RR: 3.08 | RR: 2.68 |  |  |
| 66 | Liu, 2014 | China | Glioma | Brain tissue | Normal Brain tissues | 113 | 36 | MiR-132 | up | | HR: 4.47 | HR: 6.29 |  |  |
| 67 | Men, 2014 | China | Glioma | Brain tissue | nonneoplastic Brain tissues | 266 | 266 | miR-200b | down | | HR: 2.9 | HR:4.62 |  |  |
| 68 | Sun, 2014 | China | Glioma | Brain tissue | Normal Brain tissue | 131 | 16 | MiR-155 | up | | RR: 2.639 | RR: 2.740 |  |  |
| 69 | Zhang, 2014 | China | astrocytoma | Brain tissue | Non-neoplastic Brain specimens | 122 | 30 | mir-200b | down | |  |  |  |  |
| 70 | Chen, 2015 | China | Glioma | Brain tissue | nonneoplastic Brain tissues | 137 | 137 | MiR-124 | down | | 1.562 |  |  |  |
| 71 | Cheng, 2015 | China | Glioma | Brain tissue | Normal Brain tissues | 98 | 98 | miR-218 | down | | HR: (Low vs High):3.225 |  |  |  |
| 72 | Franceschi, 2015 | Italy | GBM | Brain tissue |  | 19 |  | miRNA-150-5p |  | |  |  |  |  |
|  |  |  |  |  |  |  |  | miRNA-150-5p |  | |  |  |  |  |
|  |  |  |  |  |  |  |  | miRNA-328-3p |  | |  |  |  |  |
|  |  |  |  |  |  |  |  | miRNA-328-3p |  | |  |  |  |  |
| 73 | Guan, 2015 | China | Glioma | Brain tissue | Normal Brain tissues | 76 | 10 | miR-105 | down | | RR: (low vs. high): 4.2 |  |  |  |
| 74 | Guan, 2015 | China | Glioma | Brain tissue | Non-neoplastic Brain tissues | 63 | 10 | mir-504 | down | | RR: (low vs. high): 2.5 |  |  |  |
|  |  |  |  |  |  |  |  | miR-196a miR-367 | |  | RR: (a vs. b vs. c): 1.8 |  |  |  |
| 75 | Huang, 2015 | China | GBM | Brain tissue | Non-neoplastic Brains | 482 | 10 | miR-340 | down | |  |  |  |  |
| 76 | Wei, 2015 | China | Glioma | Brain tissue |  | 80 |  | miR-107 |  | | HR 95% CI | HR 95% CI |  |  |
| 77 | Wei, 2015 | China | Glioma | Brain tissue |  | 95 |  | miR-10b |  | | RR: 4.71 |  |  |  |
| 78 | Lai, 2015 | China | Glioma | Serum | Healthy controls | 136 | 50 | miR-210 | up | | HR: 3.84 |  |  |  |
| 79 | Wang, 2015 | China | Glioma | Brain tissue | Non-neoplastic Brain tissues | 100 | 100 | miR-378 | down | | RR: 1.68 |  |  |  |
| 80 | Guan, 2015 | China | Glioma | Brain tissue | Non-neoplastic Brain tissues | 76 | 10 | miR-15b | down | | RR: High vs. Low: 5.6 |  |  |  |
| 81 | Yan, 2015 | China | Glioma | Brain tissue |  | 92 |  | MiR-15b |  | | RR: 16.76 |  |  |  |
| 82 | Tang, 2015 | China | Glioma | Plasma | Healthy controls | 66 | 20 | MiR‑185 |  | | RR: (low vs. high): 0.179 |  | RR: 0.160 |  |
| 83 | Tong, 2015 | China | Glioma | Brain tissue | Non-neoplastic Brain tissues | 179 | 20 | miR-215 | up | |  |  |  |  |
| 84 | Wang, 2015 | China | Glioma | Brain tissue | Non-neoplastic Brain tissues | 170 | 170 | miR-214 | up | | RR: 4.78 |  |  |  |
| 85 | Xie, 2015 | China | Glioma | Brain tissue | Normal Brain tissues | 128 | 21 | miR-15a | down | |  |  |  |  |
| 86 | Xue, 2015 | China | Glioma | Brain tissue | Normal Brain tissues | 163 | 26 | miR-149 | down | | HR: 1.825 |  |  |  |
| 87 | Zhong, 2015 | China | Glioma | Brain tissue | Normal Brain tissues | 162 | 36 | miR–134 | down | | HR: 4.869 | HR: 4.269 |  |  |
| 88 | Chai, 2016 | China | Glioma | Plasma | Plasma | 55 | 55 | miR-199a-3p | down | | HR: 3.552 |  |  |  |
| 89 | Chen, 2016 | China | astrocytoma | Brain tissue | Non-neoplastic Brain tissues | 76 | 10 | miR-184 | down | | RR (Low vs. High): 5.7 |  |  |  |
| 90 | Guo, 2016 | China | Glioma | Brain tissue |  | 106 |  | Mir-326 | down | |  |  |  |  |
| 91 | Sun, 2016 | China | Glioma | Brain tissue | Non-neoplastic Brain tissues. | 92 | 13 | MiR-429 | Up | | RR:3.674 |  |  |  |
| 92 | Wang, 2016 | China | Glioma | Brain tissue | Non-neoplastic Brain tissues. | 115 | 115 | miR-154 | down | |  |  |  |  |
| 93 | Xiao, 2016 | China | Glioma | Plasma | Plasma | 112 | 54 | miR-182 | up | | HR (Low vs. high): 1.25 |  | HR (Low vs. high): 1.30 |  |
| 94 | Xue, 2016 | China | Glioma | Brain tissue | Normal Brain tissues | 87 | 18 | MiR-506 | down | | HR: 2.107 |  |  |  |
| 95 | Ye, 2016 | China | Glioma | Brain tissue | Normal Brain tissues | 105 | 10 | MiR-183 | up | | HR (High vs. low): 7.34 | HR: 7.28 |  |  |
| 96 | Yuan, 2016 | China | Glioma | Brain tissue | Non-neoplastic Brain tissues. | 116 | 15 | miR-328 | down | | mean (SE) low vs. high: 7±6 m vs. 23±9 |  |  |  |
| 97 | Zhang, 2016 | China | Glioma | Plasma | Plasma | 50 | 51 | miR-221 | up | | HR=2.40 |  |  | 0.83 |
|  |  |  |  |  |  |  |  | miR222 | up | | HR=2.81 |  |  | 0.88 |
| 98 | Zhang, 2016 | China | Glioma | Brain tissue | Normal Brain tissues | 128 | 20 | miR-10b | up | | RR: 3.284 |  |  |  |
| 99 | Zheng, 2016 | China | Glioma | Brain tissue | Non-neoplastic Brain tissues | 235 | 235 | MiR-302-3p | down | | HR: 2.54 | HR:2.92 |  |  |

# Supplementary Tables 3. Quality scores of included studies in case of Diagnosis using Newcastle-Ottawa Scale (maximum score of 9).

|  | | | | | | | | | |
| --- | --- | --- | --- | --- | --- | --- | --- | --- | --- |
| case-control studies | | | | | | | | | |
|  | Selection | | | | Comparability | outcome | | | |
| Reference | Adequate definition of cases | Representativeness of cases | Selection of controls | Definition of controls | Comparability on the basis of the design or analysis | Ascertainment of exposure | Same method of ascertainment for cases and controls | Non-response rate (<20%) | Overall |
| Baraniskin, 2012 | * | * |  | * | ** | * | * | * | 8 |
| Wang, 2012 | * | * | * | * | ** | * | * | * | 9 |
| Lai, 2015 | * | * |  | * | ** | * | * | * | 8 |
| Sun, 2015 | * | * | * | * | ** | * | * | * | 9 |
| Chai, 2015 | * | * | * | * | ** | * | * | * | 9 |
| Xiao, 2016 | * | * | * | * | ** | * | * | * | 9 |
| Yue, 2016 | * | * | * | * | ** | * | * | * | 9 |
| Zhao,2016 | * | * |  | * | ** | * | * | * | 8 |
| Huang, 2017 | * | * |  | * | ** | * | * | * | 8 |
| Tang, 2017 | * | * |  | * | ** | * | * | * | 8 |
| Xu, 2017 | * | * |  | * | ** | * | * | * | 8 |
| Lan, 2018 | * | * |  | * | ** | * | * | * | 8 |
| Santangelo, 2018 | * | * |  | * | ** | * | * | * | 8 |
| Kopkova, 2019 | * | * |  | * |  | * | * | * | 6 |
| Ohno, 2019 | * | * |  | * | ** | * | * | * | 8 |
| Wang, 2019 | * | * |  | * | ** | * | * | * | 8 |
| Zhang, 2019 |  |  |  | * | ** | * | * | * | 6 |
| ZHUm 2019 | * | * |  | * | ** | * | * | * | 8 |
| Chen, 2020 | * | * |  | * | ** | * | * | * | 8 |
| Lan, 2020 | * | * |  | * | ** | * | * | * | 8 |
| Sun, 2021 | * | * |  | * | ** | * | * | * | 8 |
| Catelan, 2022 |  |  |  | * | ** | * | * | * | 6 |
| Geng, 2022 |  |  |  | * | ** | * | * | * | 6 |
| Nikolova, 2022 |  |  |  | * | ** | * | * | * | 6 |
| Wu, 2022 | * | * | * | * | ** | * | * | * | 9 |

| Supplementary Table 4. Quality scores of included studies in case of Prognosis using Newcastle-Ottawa Scale (maximum score of 9). | | | | | | | | | |
| --- | --- | --- | --- | --- | --- | --- | --- | --- | --- |
| case-control studies | | | | | | | | | |
|  | Selection | | | | Comparability | outcome | | | |
| Reference | Adequate definition of cases | Representativeness of cases | Selection of controls | Definition of controls | Comparability on the basis of the design or analysis | Ascertainment of exposure | Same method of ascertainment for cases and controls | Non-response rate (<20%) | Overall |
| Bai, Q 2017 | * | * |  | * | ** | * | * | * | 8 |
| chen,J 2017 | * | * |  | * | ** | * | * | * | 8 |
| cheng, z,x, 2017 | * | * |  | * | ** | * | * | * | 8 |
| Hua, Z, 2017 | * | * |  |  | ** | * |  |  | 5 |
| Jing, S, Y, 2017 | * | * |  |  | ** | * |  |  | 5 |
| Li, H, 2017 | * | * |  | * | ** | * | * | * | 8 |
| Lv, Q, L, 2017 | * | * |  | * | ** | * | * | * | 8 |
| Qin, C, Z, 2017 | * | * |  | * | ** | * | * | * | 8 |
| Qin, W, 2017 | * | * |  | * | ** | * | * | * | 8 |
| Tang, Y, 2017 |  | * | * | * | ** | * | * | * | 8 |
| Xiao, Z, Q, 2017 | * | * |  | * | ** | * | * | * | 8 |
| Xue, L, 2017 | * | * |  | * | ** | * | * | * | 8 |
| Yan,H, 2017 | * | * |  | * | ** | * | * | * | 8 |
| Yang, M, 2017 | * | * |  |  | ** | * |  |  | 5 |
| Ye, Z, n, 2017 | * | * |  |  | ** | * |  |  | 5 |
| Yuan, G, Q, 2017 | * | * |  |  | ** | * |  |  | 5 |
| Zhang, J, 2017 | * | * |  |  | ** | * |  |  | 5 |
| Chen, Y, Y, 2018 | * | * |  |  | ** | * |  |  | 5 |
| Lan, F, 2018 | * | * | * | * | ** | * | * | * | 9 |
| LV, Q, l, 2018 | * | * |  | * | ** | * | * | * | 8 |
| Wang, H, 2018 | * | * |  |  | ** | * |  |  | 5 |
| Xue, K, 2018 | * | * |  | * | ** | * | * | * | 8 |
| Zhou, X, Y, 2018 | * | * |  |  | ** | * |  |  | 5 |
| Chen, Y, 2019 | * | * |  | * | ** | * | * | * | 8 |
| Dong, Q, 2019 | * | * |  | * | ** | * | * | * | 8 |
| Feng, F, Q, 2019 | * | * |  | * | ** | * | * | * | 8 |
| Sippl, C., et al. ,2019 | * | * |  |  | ** | * |  |  | 5 |
| Song, Q., et al. ,2019 | * | * |  | * | ** | * | * | * | 8 |
| Stupak, E. V., et al. (2019 | * | * |  | * | ** | * | * | * | 8 |
| Wang, J. F., et al. ,2019 |  | * | * | * | ** | * | * | * | 8 |
| Wang, L. Q., et al. ,2019 | * | * | * | * | ** | * | * | * | 9 |
| Wang, W, 2019 | * | * |  | * | ** | * | * | * | 8 |
| Wu, M., et al. ,2019 | * | * |  | * | ** | * | * | * | 8 |
| Xiao, H., et al. ,2019 | * | * |  |  | ** | * |  |  | 5 |
| Zhang, Y., et al., 2019 |  |  |  | * | ** | * | * | * | 6 |
| Zhu, M., et al., 2019 | * | * |  | * | ** | * | * | * | 8 |
| Chen, J., et al., 2020 |  | * |  |  | ** | * | * | * | 6 |
| Chen, P., et al., 2020 | * | * |  | * | ** | * | * | * | 8 |
| Ji, Y., et al., 2020 | * | * |  |  | ** | * |  |  | 5 |
| Lan, F., et al. (2020) | * | * | * | * | ** | * | * | * | 9 |
| Morokoff, A., et al. (2020) | * | * | * | * | ** | * | * | * | 9 |
| Kai, Q., et al. ,2021 | * | * |  | * | ** | * | * | * | 8 |
| Li, Y., et al. ,2021 | * | * |  | * | ** | * | * | * | 8 |
| Liu, P., et al. ,2021 | * | * |  |  | ** | * |  |  | 5 |
| Qu, S., et al. ,2021 | * | * |  |  | ** | * |  |  | 5 |
| Sun, J., et al. ,2021 | * | * |  | * | ** | * | * | * | 8 |
| Wang, H., et al. ,2021 |  | * |  |  |  | * |  |  | 2 |
| Xiao, F., et al. ,2021 |  | * |  |  | ** | * |  |  | 4 |
| Zheng, H., et al. ,2021 | * | * |  | * | ** | * | * | * | 8 |
| Wei, G., et al. (2022). | * | * |  | * | ** | * | * | * | 8 |
| Li, 2013 | * | * |  | * | ** | * | * | * | 8 |
| Lu, 2013 | * | * |  | * | ** | * | * | * | 8 |
| Parker, 2013 | * | * |  | * | ** | * | * | * | 8 |
| Zhao, 2013 | * | * |  |  | ** | * |  |  | 5 |
| Barbano, 2014 | * | * |  | * | ** | * | * | * | 8 |
| Lai, 2014 | * | * |  | * | ** | * | * | * | 8 |
| Men, 2014 | * | * |  | * | ** | * | * | * | 8 |
| Sun, 2014 | * | * |  | * | ** | * | * | * | 8 |
| Jiang,L, 2010 | * | * |  | * | ** | * | * | * | 8 |
| Costa,F, 2011 | * | * |  | * | ** | * | * | * | 8 |
| Chang, C, 2012 | * | * |  | * | ** | * | * | * | 8 |
| Lu, S, 2012 | * | * |  | * | ** | * | * | * | 8 |
| Wu, Z, 2012 | * | * |  |  |  |  |  |  | 2 |
| Gao, H, 2013 | * | * |  | * | ** | * | * | * | 8 |
| Harmansen, S, 2013 | * | * |  |  | ** | * |  |  | 5 |
| Delfino, 2011 |  | * |  |  | ** | * |  |  | 4 |
| Srinivasan, 2011 |  | * |  |  | ** | * |  |  | 4 |
| Lu, 2013 | * | * |  | * | ** | * | * | * | 8 |
| Li, 2013 | * | * |  | * | ** | * | * | * | 8 |
| Parker, 2013 | * | * |  | * | ** | * | * | * | 8 |
| Liu, 2014 | * | * |  | * | ** | * | * | * | 8 |
| Zhang, 2014 | * | * |  | * | ** | * | * | * | 8 |
| Chen, 2015 | * | * |  | * | ** | * | * | * | 8 |
| Cheng, 2015 | * | * |  | * | ** | * | * | * | 8 |
| Franceschi, 2015 | * | * |  | * | ** | * | * | * | 8 |
| Guan, 2015 | * | * |  | * | ** | * | * | * | 8 |
| Guan, 2015 | * | * |  | * | ** | * | * | * | 8 |
| Huang, 2015 | * | * |  | * | ** | * | * | * | 8 |
| Wei, 2015 | * | * |  |  | ** | * |  |  | 5 |
| Wei, 2015 | * | * |  |  | ** | * |  |  | 5 |
| Lai, 2015 | * | * |  | * | ** | * | * | * | 8 |
| Wang, 2015 | * | * |  | * | ** | * | * | * | 8 |
| Guan, 2015 | * | * |  | * | ** | * | * | * | 8 |
| Yan, 2015 | * | * |  |  | ** | * |  |  | 5 |
| Tang, 2015 | * | * |  | * | ** | * | * | * | 8 |
| Tong, 2015 | * | * |  | * | ** | * | * | * | 8 |
| Wang, 2015 | * | * |  | * | ** | * | * | * | 8 |
| Xie, 2015 | * | * |  | * | ** | * | * | * | 8 |
| Xue, 2015 | * | * |  | * | ** | * | * | * | 8 |
| Zhong, 2015 | * | * |  | * | ** | * | * | * | 8 |
| Chai, 2016 | * | * |  | * | ** | * | * | * | 8 |
| Chen, 2016 | * | * |  | * | ** | * | * | * | 8 |
| Guo, 2016 | * | * |  |  | ** | * |  |  | 5 |
| Sun, 2016 | * | * |  | * | ** | * | * | * | 8 |
| Wang, 2016 | * | * |  | * | ** | * | * | * | 8 |
| Xiao, 2016 | * | * | * | * | ** | * | * | * | 9 |
| Xue, 2016 | * | * |  | * | ** | * | * | * | 8 |
| Ye, 2016 | * | * |  | * | ** | * | * | * | 8 |
| Yuan, 2016 | * | * |  | * | ** | * | * | * | 8 |
| Zhang, 2016 | * | * |  | * | ** | * | * | * | 8 |
| Zhang, 2016 | * | * |  | * | ** | * | * | * | 8 |
| Zheng, 2016 | * | * |  | * | ** | * | * | * | 8 |

# Supplementary Figure 1. Summary ROC curve comparing different types of specimens.
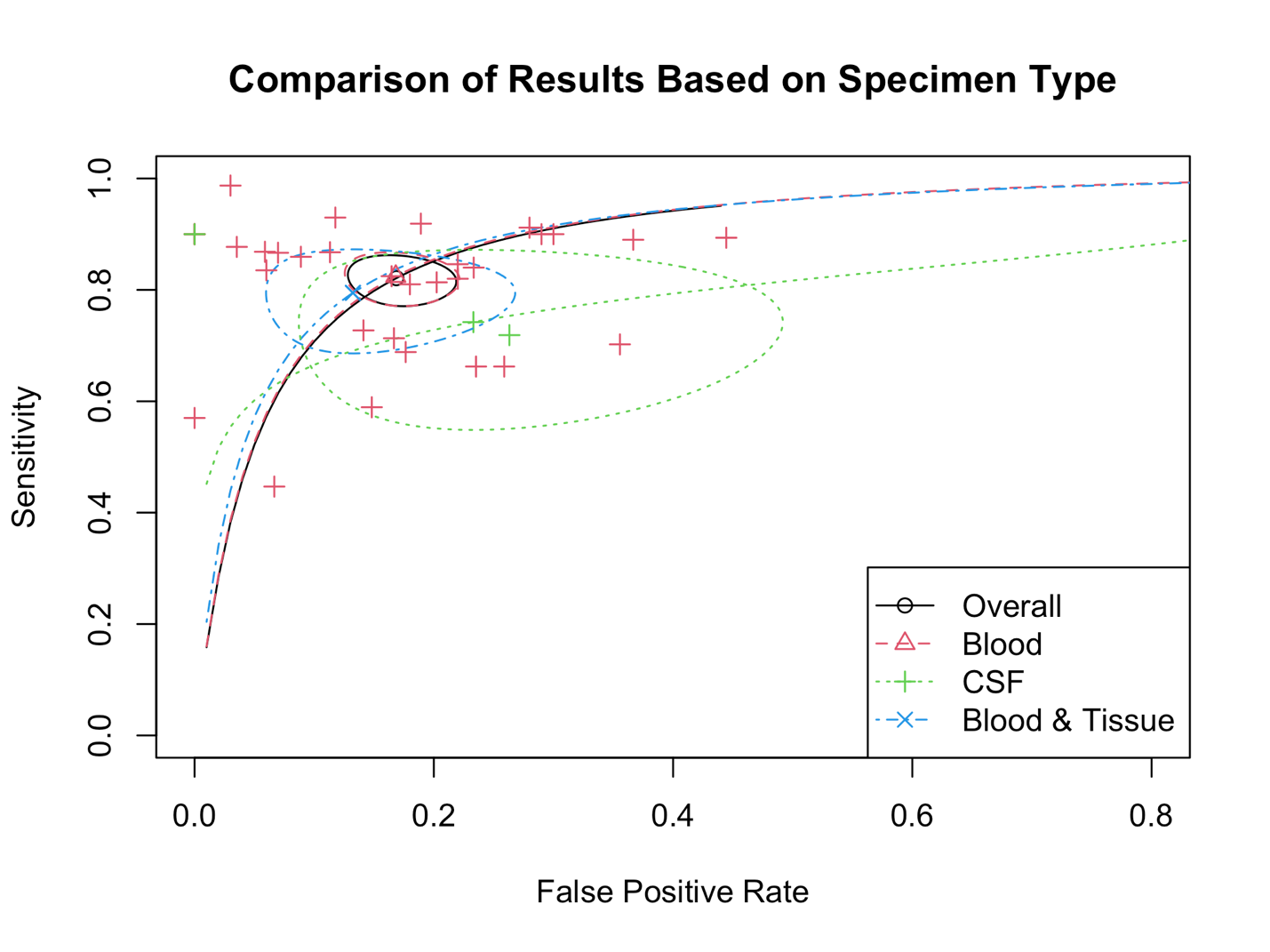


# Supplementary Figure 2. Meta-analysis of quality assessment + specimen subgrouping for AUCs.

**
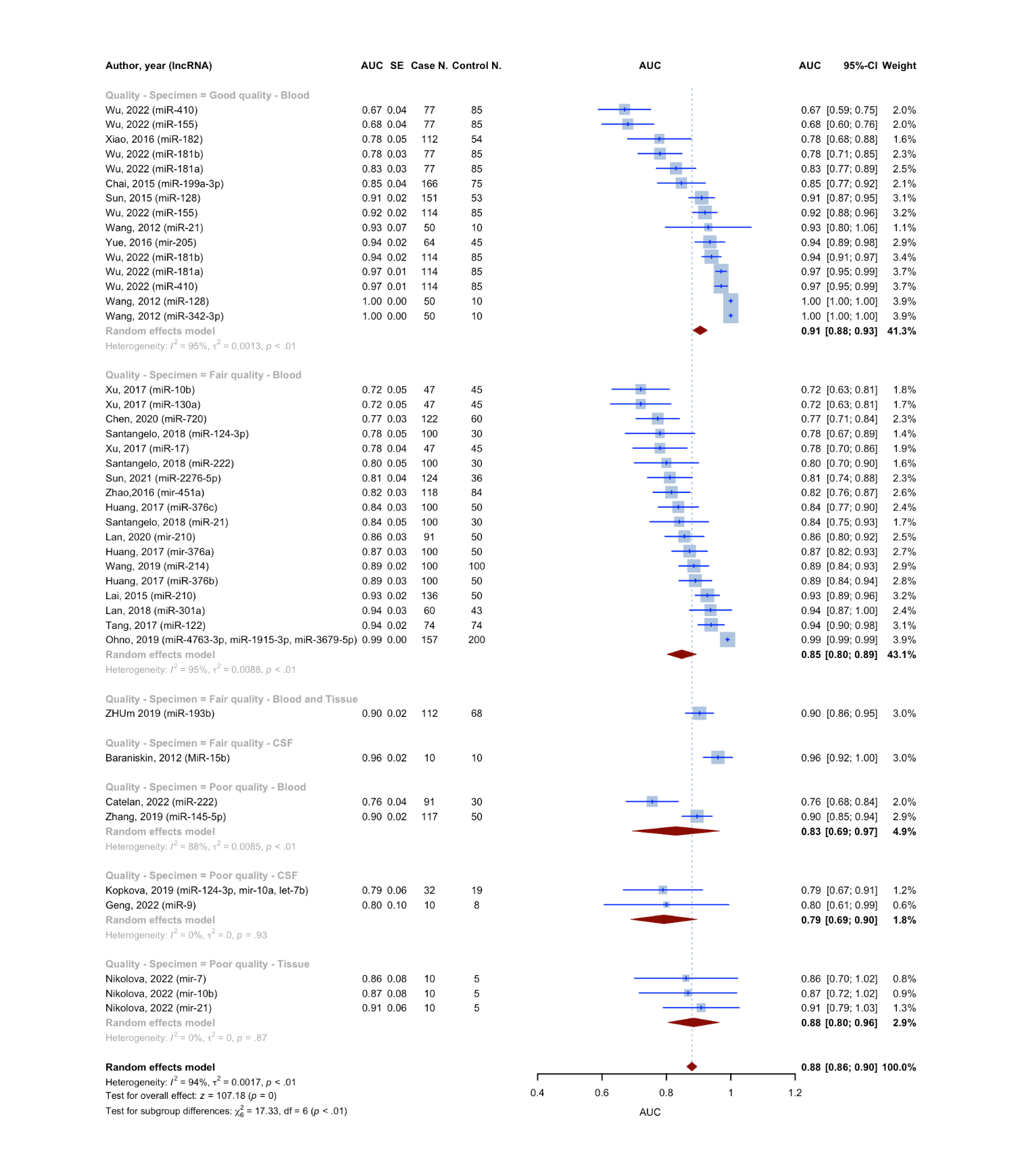
**

# Supplementary Figure 3. Meta-analysis of quality assessment + specimen subgrouping for overall survival hazard ratios.


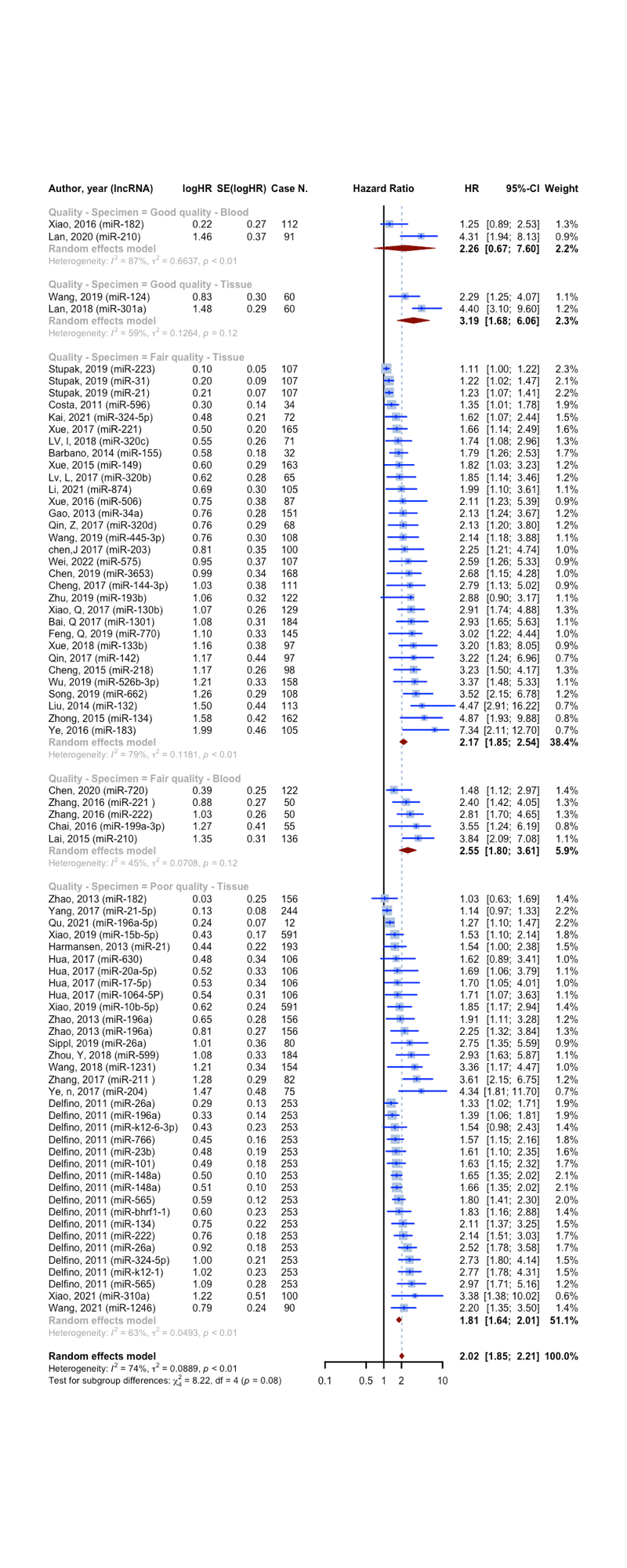

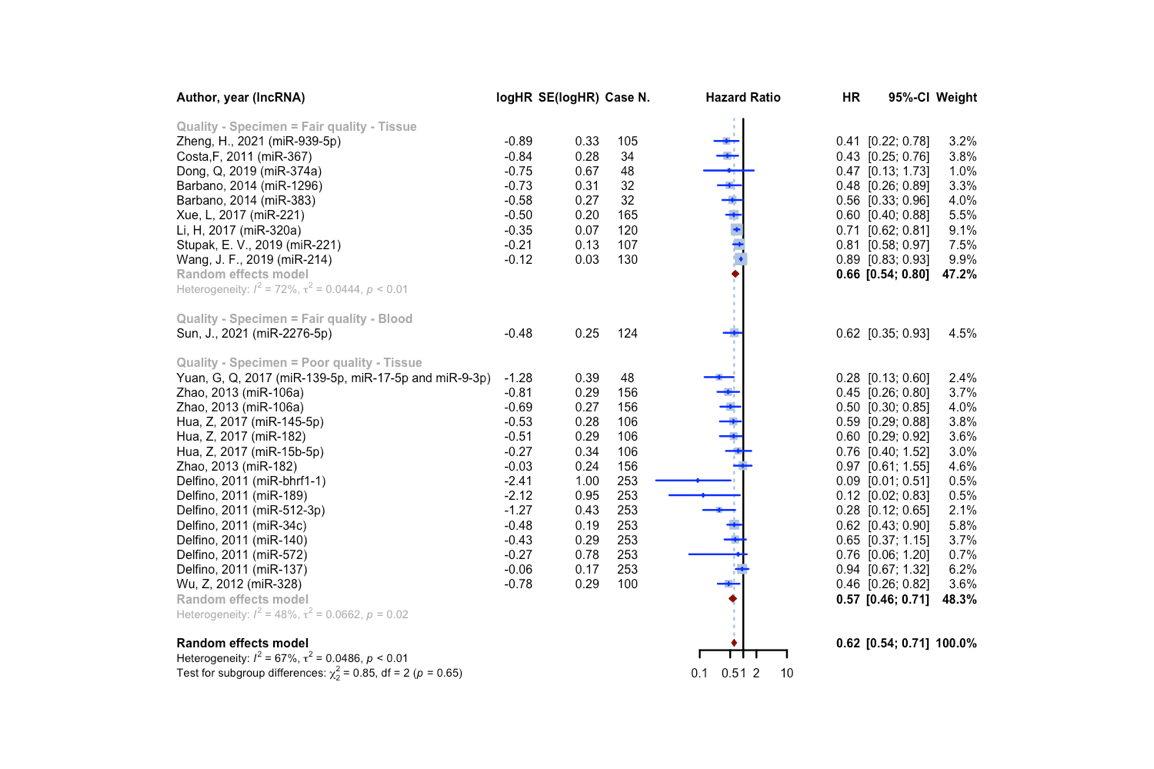


# Supplementary Figure 4. Meta-analysis of quality assessment + specimen subgrouping for progression-free survival hazard ratios.


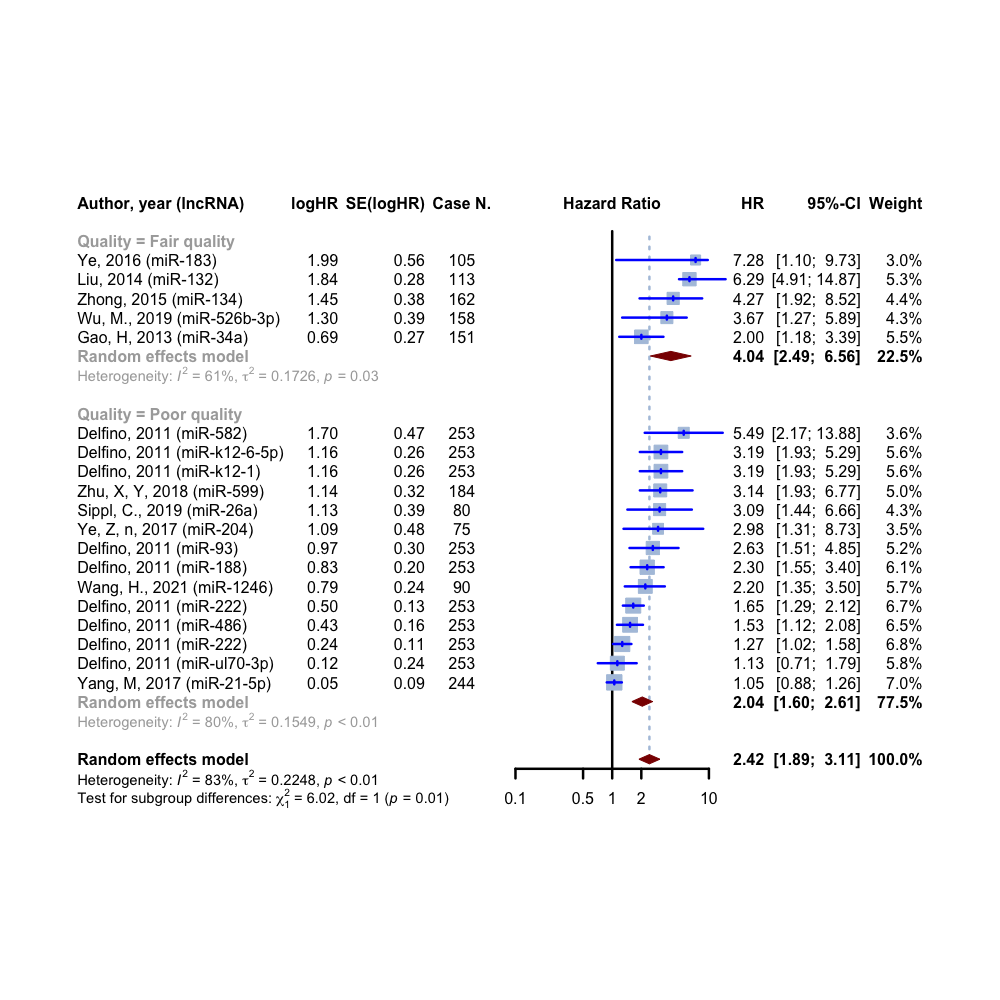

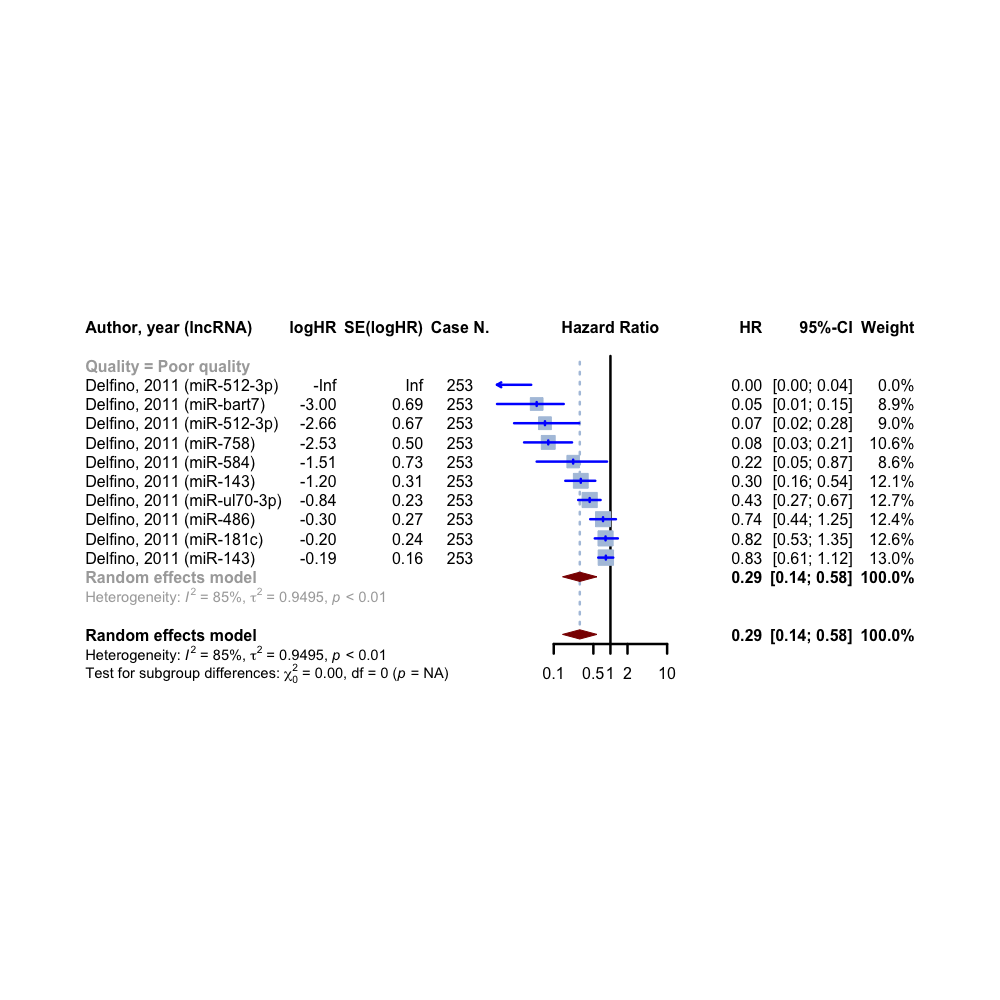


# Supplementary Figure 5. The disease-free survival hazard ratios meta-analysis.


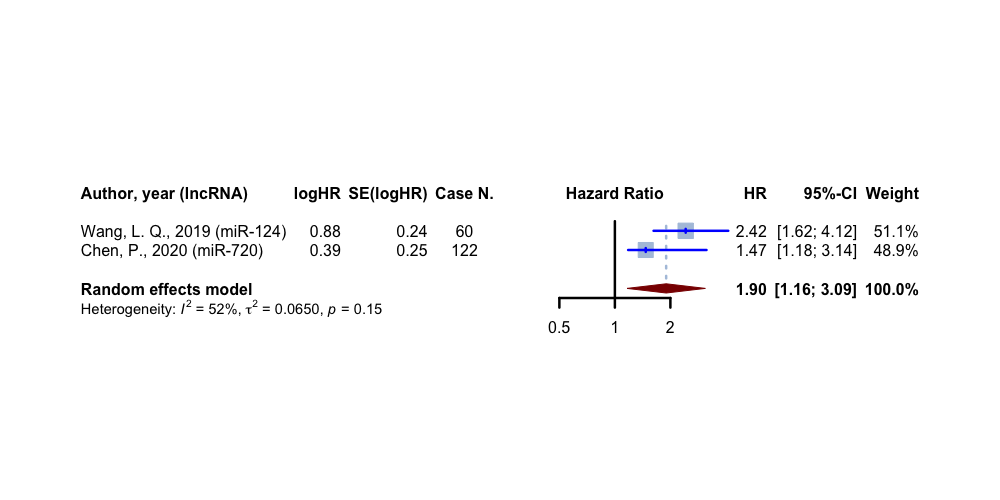

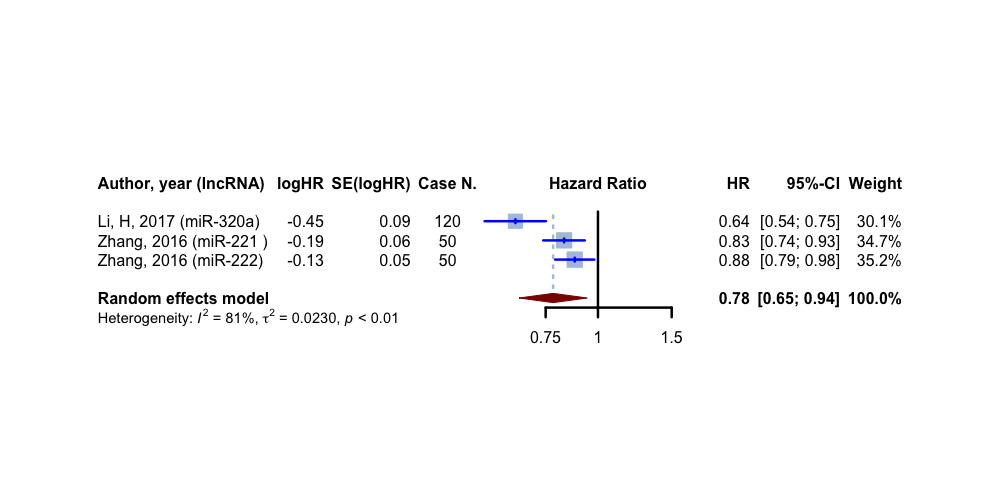


**
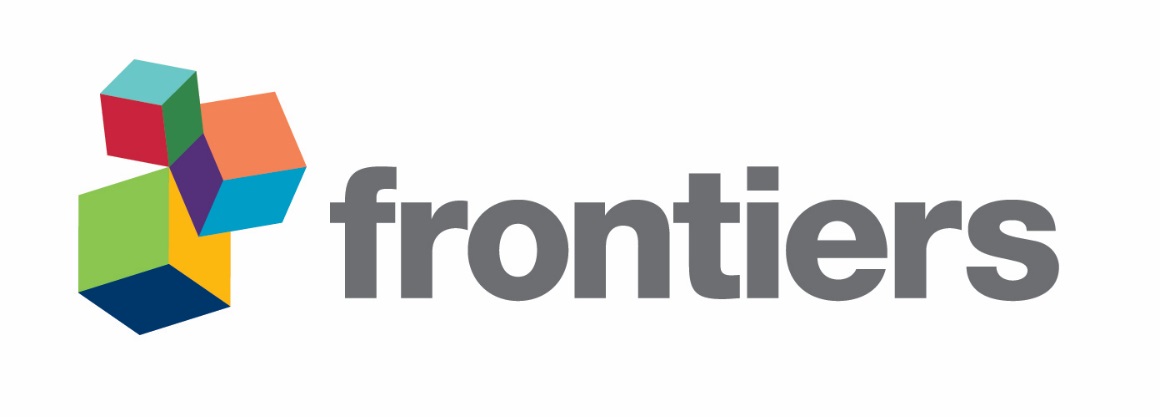
**

**Supplementary Figure 1.** The figure legends are required to have the same font as the main text, 12 point normal Times New Roman, single spaced. Please use a single paragraph for each legend and prepare the figures keeping in mind the PDF layout.
